# The *Thermoanaerobacter* Glycobiome Reveals Mechanisms of Pentose and Hexose Co-Utilization in Bacteria

**DOI:** 10.1371/journal.pgen.1002318

**Published:** 2011-10-13

**Authors:** Lu Lin, Houhui Song, Qichao Tu, Yujia Qin, Aifen Zhou, Wenbin Liu, Zhili He, Jizhong Zhou, Jian Xu

**Affiliations:** 1 CAS Key Laboratory of Biofuels, Shandong Key Laboratory of Energy Genetics and BioEnergy Genome Center, Qingdao Institute of BioEnergy and BioProcess Technology, Chinese Academy of Sciences, Qingdao, China; 2 Institute for Environmental Genomics and Department of Botany and Microbiology, University of Oklahoma, Norman, Oklahoma, United States of America; Universidad de Sevilla, Spain

## Abstract

Thermoanaerobic bacteria are of interest in cellulosic-biofuel production, due to their simultaneous pentose and hexose utilization (co-utilization) and thermophilic nature. In this study, we experimentally reconstructed the structure and dynamics of the first genome-wide carbon utilization network of thermoanaerobes. The network uncovers numerous novel pathways and identifies previously unrecognized but crucial pathway interactions and the associated key junctions. First, glucose, xylose, fructose, and cellobiose catabolism are each featured in distinct functional modules; the transport systems of hexose and pentose are apparently both regulated by transcriptional antiterminators of the BglG family, which is consistent with pentose and hexose co-utilization. Second, glucose and xylose modules cooperate in that the activity of the former promotes the activity of the latter via activating xylose transport and catabolism, while xylose delays cell lysis by sustaining coenzyme and ion metabolism. Third, the vitamin B_12_ pathway appears to promote ethanologenesis through ethanolamine and 1, 2-propanediol, while the arginine deiminase pathway probably contributes to cell survival in stationary phase. Moreover, by experimentally validating the distinct yet collaborative nature of glucose and xylose catabolism, we demonstrated that these novel network-derived features can be rationally exploited for product-yield enhancement via optimized timing and balanced loading of the carbon supply in a substrate-specific manner. Thus, this thermoanaerobic glycobiome reveals novel genetic features in carbon catabolism that may have immediate industrial implications and provides novel strategies and targets for fermentation and genome engineering.

## Author Summary

Renewable liquid fuels derived from lignocellulosic biomass could alleviate global energy shortage and climate change. Cellulose and hemicellulose are the main components of lignocellulosic biomass. Therefore, the ability to simultaneously utilize pentose and hexose (i.e., co-utilization) has been a crucial challenge for industrial microbes producing lignocellulosic biofuels. Certain thermoanaerobic bacteria demonstrate this unusual talent, but the genetic foundation and molecular mechanism of this process remain unknown. In this study, we reconstructed the structure and dynamics of the first genome-wide carbon utilization network of thermoanaerobes. This transcriptome-based co-expression network reveals that glucose, xylose, fructose, and cellobiose catabolism are each featured on distinct functional modules. Furthermore, the dynamics of the network suggests a distinct yet collaborative nature between glucose and xylose catabolism. In addition, we experimentally demonstrated that these novel network-derived features can be rationally exploited for product-yield enhancement via optimized timing and balanced loading of the carbon supply in a substrate-specific manner. Thus, the newly discovered modular and precisely regulated network elucidates unique features of thermoanaerobic glycobiomes and reveals novel perturbation strategies and targets for the enhanced thermophilic production of lignocellulosic biofuels.

## Introduction

Renewable liquid fuels derived from lignocellulose, the most abundant biological polymer on earth, could alleviate global energy shortages and climate change. Consolidated bioprocessing (CBP), which is one proposed scheme of lignocellulosic ethanol production, combines cellulase production, cellulose degradation, hexose fermentation and pentose fermentation in a single bioreactor, thus maximizing energy- and cost-saving [1]. Thermophilic, gram-positive, anaerobic bacteria (TGPAs) are of exceptional interest in a CBP scheme, due to several advantages, including the capability of rapid cellulose degradation (e.g., *Clostridium thermocellum*) [2], the ability to ferment a wide range of monosaccharides and oligosaccharides (e.g., *Thermoanaerobacter*), and optimal growth at high temperature (60–70°C), which avoids iterative heating/cooling steps, saves energy for downstream product recovery and minimizes microbial contamination [2]. Furthermore, TGPAs, such as *Thermoanaerobacter*, not only metabolize both hexose and pentose but also simultaneously ferment them into ethanol (“co-utilization”). This feature is of particular industrial interest because the pentose D-xylose is the primary ingredient of the hemicellulose fraction of lignocellulosic biomass.

However, optimization of TGPA cellular machineries for cellulosic ethanol production through fermentation or genetic engineering has been a challenge. First, continuous ethanol production is highly dependent on the efficient and simultaneous use of all of the di- and monosaccharides (both pentoses and hexoses) released from lignocellulose. Second, along with ethanologenic pathways, all described TGPAs have branched organic acid pathways [3]. Currently, the ethanol yield (typically <2%) [3], carbon substrate loading (e.g., <27 g/L cellulose for *C. cellulolyticum* and <20 g/L xylose for *T. ethanolicus* [4]), ethanol tolerance (usually <1∼1.5% (w/v) [5]) and sugar to ethanol conversion rate (usually <30%) [3] have hindered the direct industrial application of TGPAs.

Genetic engineering of TGPA ethanologens has focused thus far on knocking out individual enzyme genes in the acetate and lactate pathways [3]. However, the highest yield reported (3.7% (w/v)) is still far below the industrial demand (7% or higher) [1]. Alternatively, few studies have engineered carbon-influx machineries in which shared or specific catabolic pathways of various carbohydrate substrates interact to mediate the carbon flux in the cell. In organisms such as *Escherichia coli* and *Bacillus subtilis*, the catabolism of monosaccharides and oligosaccharides is tightly controlled by key regulatory processes, such as carbon catabolite repression (CCR), resulting in a cellular preference for hexoses over pentoses [6]. However, little is known about how the unusual phenotype of hexose-pentose co-utilization found in some TGPAs occurs, and few genome-wide models of thermoanaerobic carbohydrate catabolism (“thermoanaerobic glycobiomes”) have been reported [7].

Employing the thermophilic ethanologen *Thermoanaerobacter* sp. X514 as a model [8], [9], we devised a rational strategy to unravel the structure and dynamics of the thermoanaerobic glycobiome. Whole-genome expression profiles and physiological responses at various growth phases were measured while culturing X514 in glucose, xylose, fructose and cellobiose as sole carbon sources or in pairwise combinations. Diversity, interactions and dynamics of functional modules were identified and interrogated via co-expression analysis and comparative genomics. To the best of our knowledge, these efforts enabled the reconstruction of the first genome-wide functional network operating and regulating a thermoanaerobic glycobiome.

## Results

### *Thermoanaerobacter* glycobiome network simultaneously metabolizes hexose and pentose

**Figure 1:**
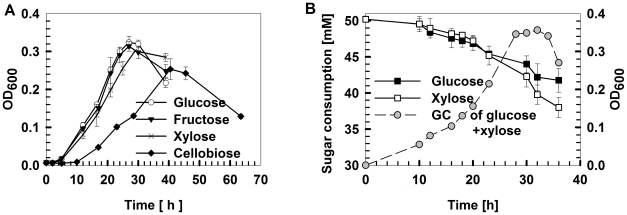
*Thermoanaerobic*growth conditions and carbohydrate utilization by*Thermoanaerobacter*sp. X514. The strain was grown in defined medium supplemented with glucose, xylose, fructose or cellobiose as the sole carbon source or in pairwise combinations. A) Growth curves under different mono-carbohydrates. B) Time course of sugar utilization when grown in defined medium supplemented with 50 mM glucose plus 50 mM xylose. All experiments were performed in triplicate. GC: growth curve.

**Figure 2:**
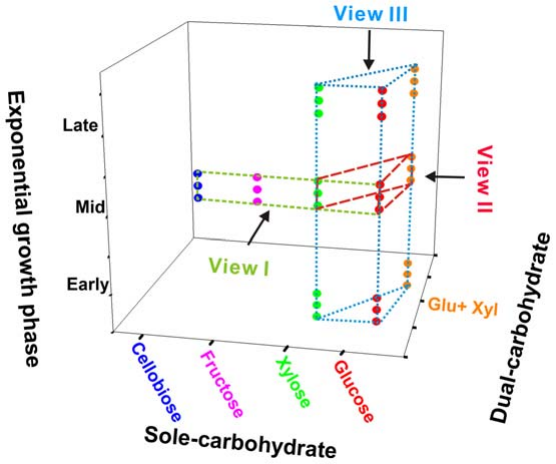
Experimental design for reconstructing the gene co-expression network. View I investigated four carbon substrate-specific cellular machineries. View II explored the interactions between hexose and pentose catabolism. View III captured the network dynamics at different growth phases. Three dots under every condition represent the three biological replicates. Whole-genome expression profiles of all 33 samples were used to reconstruct the gene co-expression network.

**Figure 3:**
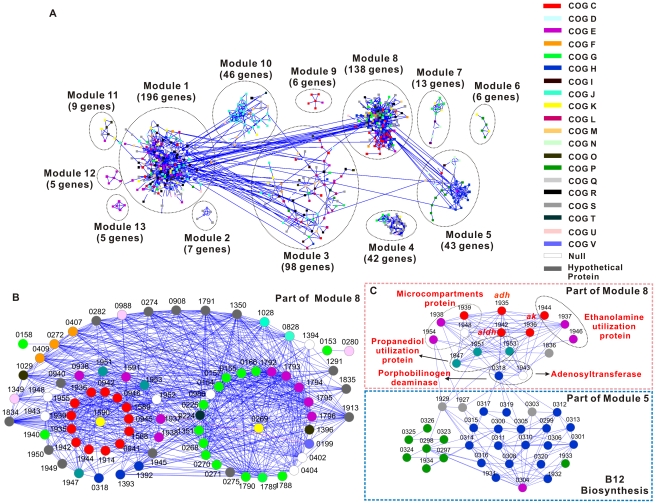
Gene co-expression network of the *Thermoanaerobacter* glycobiome. A) Global view of the network. B) A sub-module of Module 8. Only those genes known to be involved in xylose catabolism and their first neighbors (genes directly connected to xylose catabolism genes) in the network are shown. C) The sub-network that includes genes directly involved in B_12_ synthesis and ethanol fermentation. It includes components from both Module 5 and Module 8. Each node represents a gene, which is color-coded using its predicted COG-based functional classification (http://www.ncbi.nlm.nih.gov/nuccore/NC_010320; NC_010320.1). Any two genes with a Pearson correlation ≥0.94 in their expression patterns are connected (via edges, which are shown as lines; blue lines indicate positive correlation). For a given gene, its first neighbors are defined as those gene(s) that are directly connected to it.

Among the glucose, xylose, fructose and cellobiose carbon sources, X514 more rapidly and efficiently catabolized monosaccharides than disaccharides (Figure 1A and Figure S1E). Moreover, different carbon substrates resulted in distinct product sets (Part I of Text S1). However, X514 simultaneously and efficiently metabolized both hexose and pentose when both were present, suggesting an absence of CCR (Figure 1B). To investigate the cause of this phenomenon, a high-density, oligonucleotide-based, whole-genome, gene-expression microarray for X514 was constructed and analyzed via two-dimensional transcriptome sampling (Figure 2): one by carbon substrate (glucose, xylose, fructose and cellobiose) and the other by growth phase (early, mid and late exponential). The sampling and analyses were further organized into three “Views” (Figure 2). View I investigated carbon substrate-specific cellular machineries where X514 transcriptomes of mono-carbohydrate cultures (glucose, xylose, fructose or cellobiose alone) were collected and compared at mid exponential phase. View II was designed to explore the interactions between the hexose and pentose pathways where mid exponential cultures under glucose alone, xylose alone and the equimolar presence of glucose and xylose (glucose-xylose) were collected and investigated. In View III, network dynamics were captured by sampling early, mid and late exponential phase cultures under different substrates (glucose alone, xylose alone or glucose-xylose). All of these experiments were performed with three biological replicates to improve the discriminating power of the subsequent co-expression analyses. A genome-wide functional network consequently emerged.

(I) Overview of the Network. The network includes a total of 614 genes (24.8% of the genome) that are partitioned into thirteen modules (Figure 3A and Table S1). Each module represents a group of more than four genes that are highly connected among themselves but have fewer connections with those in other modules [10]. Thus, each module consists of a functionally coherent set of genes (‘nodes’) and a set of connections (‘links’) that suggest positive co-expression (i.e., functional correlation) between the two nodes.

Module 1 (mostly genes involved in amino acid metabolism, translation, transcription or encoding hypothetical proteins), Module 3 (mostly genes related to DNA replication or encoding hypothetical proteins), Module 8 (mostly xylose catabolism and energy production genes) and Module 10 (mostly 30S or 50S ribosomal protein genes) are the most predominant (Figure 3A), suggesting their contribution as the core sugar-responsive pathways in X514. Modules 5 (mostly genes involved in *de novo* vitamin B_12_ synthesis and related cobalt transport), Module 6 (genes responsible for heavy metal transport), Module 9 (mostly V-type ATP synthesis genes) and Module 11 (mostly genes involved in chaperones and stress responses) also are identified, although they are generally much smaller in size (Figure 3A). Furthermore, distinct modules that correspond to xylose, fructose and cellobiose utilization are revealed (Module 8, 7 and 4 respectively; Figure 3A).

(II) Structures of Individual Modules. The network serves as an experimental basis for unraveling, validating and annotating the structure and function of “hypothetical genes”, operons and regulatory circuits (Part II of Text S1; Figure S2, Figure S3 and Table S2). Because there are 13 prominent modules in the network, only Module 8, which is responsible for xylose catabolism, is presented here as an example.

Module 8 includes the majority of genes in xylose catabolism and includes 138 nodes (genes) and 2001 links. Three major Clusters of Orthologous Groups (COG) categories exist in this module: carbohydrate transport and metabolism (COG G, 19 genes), energy production and conversion (COG C, 19 genes) and amino acid transport and metabolism (COG E, 12 genes) (Figure 3B). These 50 genes account for 36% of all genes in this module.

Genes in COG G occupy two groups, one of which is the xylose-specific monosaccharide ATP-binding cassette (ABC) transporter systems (*teth5140155* (*xylF*), *0157-0158* (*xylGH*), *0166*, *0225* and *0989*). Similar to *Thermoanaerobacter ethanolicus* 39E, which is a strain closely related to X514, and *E. coli*, the xylose ABC transporter of X514 consists of three molecular components: an ATP-binding protein (XylG, Teth5140157), a membrane transporter (XylH, Teth5140158) and a substrate-binding protein (XylF, Teth5140155) (*teth5140156* encodes an RNA-directed DNA polymerase) [11], [12]. However, the organization of these genes in the X514 genome is distinct from in *E. coli* and 39E (Figure S4). In X514, *xylF* and *xylAB* (encoding xylose metabolic enzymes) are in one putative operon, whereas *xylGHR* resides in another locus located downstream of *xylABF*. Moreover, in X514, the *xylABF* and *xylGH* are both upregulated under xylose (Table S3), while in 39E the expression of *xylH* is relatively constant [11], regardless of xylose. Thus, X514 is unique in that its xylose ABC transporters are xylose-dependent. The other group includes genes encoding xylose metabolic enzymes (*teth5140154* (*xylB*), *0969* and *1351*), which are strongly induced under xylose (Table S3). Interestingly, *xylA* (*teth5140153*; Module 1), a member of one Module 8 predicted operon (*teth5140153-0155*), serves as the connection between an ATPase gene (*teth5140280*; Module 1) and the rest of the putative operon, thus identifying Teth5140280 as the ATP provider for xylose transport and initial metabolism (Figure 3B).

The COG C genes in Module 8 include two predicted operons related to butanoate metabolism (*teth5140936-0938* and *teth5140939-0947*), two loci involved in the ethanolamine utilization pathway (*teth5141937-1939* and *teth5141943-1944*), acetate kinase (*teth5141936*) and alcohol dehydrogenase (*teth5141935*). These genes, all of which are key participants of energy (ATP) and ethanol generation, are upregulated under xylose (Table S4). Therefore, xylose supports a vigorous ethanologenic program in X514.

The COG E genes in Module 8 mainly occupy two putative operons: one encoding oligopeptide/dipeptide ABC transporters (*teth5141792-1796*) and the other for ethanolamine utilization proteins (*teth5141945-1946*). In fact, genes involved in vitamin B_12_-dependent ethanolamine utilization, including those in COG C and COG E, are all grouped into this module. Moreover, they are all upregulated under xylose (Table S5). Ethanolamine produces acetaldehyde, which is subsequently converted to acetate and ethanol by aldehyde dehydrogenase (Aldh; Teth5141942) and alcohol dehydrogenase (Adh; Teth5141935) (Figure 3C and Figure S5D). Consistent with this, microcompartment proteins (Teth5141938-1939), which protect cytosolic proteins from aldehyde toxicity [13], are also found in Module 8 and are highly upregulated, representing a potential cellular detoxification mechanism against aldehydes in X514.

Aside from the three major COG categories, an additional 54 genes (39.1% of total) are found in the Module 8, including several newly recognized sub-modules, such as ABC transporters and the B12-dependent propanediol utilization pathway (Part III of Text S1; Figure S3). The most notable is a regulatory sub-module centering on the transcriptional antiterminator bglG (teth5140269) in the putative mannitol-specific phosphotransferase system (PTS) operon (Figure S3A). In the network, the bglG is directly linked to the other members of this locus (*teth5140268-0270*), suggesting that it regulates their expression. Surprisingly, the *bglG* is also positively linked to the genes encoding oligopeptide/dipeptide (*teth5141792-1796*) and xylose (*teth5140157*) ABC transporters. However, no links are found between the xylose transporters (*teth5140155*-*0157*) and the Module 1 *xylR* (*teth5140159*), which encodes the transcriptional factor regulating xylose transporters in other bacteria, including *E. coli* [12], [14] (Figure S3B). Moreover, the expression of *bglG* but not *xylR* is induced under xylose (Table S3). These data suggest that, in response to xylose availability, BglG regulates xylose transport (instead of XylR) via activation of the putative operons in X514.

In addition to Modules 8, 7 and 4, which are involved in xylose, fructose and cellobiose utilization respectively (Part IV and Part V of Text S1; Figure S2B, Figure S5A and Tables S6, S7, S8, S9, S10), the network unravels previously unrecognized functional modules in the X514 glycobiome (Figure 3A; Part VI of Text S1). For example, the presence of Module 5 demonstrates *de novo* B_12_ cofactor biosynthesis in X514, which simplifies nutrient requirements for cells, and reveals the molecular link between B_12_ and carbon metabolism. Additionally, the crucial role of arginine metabolism in the glycobiome is revealed by the discovery of Module 13, which includes arginine metabolism genes (Figure 3A). Arginine serves as a precursor for additional ATP and NH_3_ via the arginine deiminase (ADI) pathway to protect the cell against damage caused by energy depletion and acidification [15]. Under xylose, arginine (*argABCDFGH*, *teth5140657-0663*) and glutamate biosynthesis (the precursor of arginine biosynthesis; *teth5140505* and *teth5140468-0470*) genes are highly expressed (Table S5) during mid exponential phase. Furthermore, the ADI pathway locus (*teth5140483*-*0485*) is activated during late exponential phase under xylose. Therefore, we propose that, in response to xylose, arginine is actively synthesized during mid exponential phase, which could serve as an alternative energy source for ATP generation during late exponential or stationary phases, consistent with the observation that X514 had a longer stationary phase under xylose than glucose (Figure 1A). How xylose activates the ADI pathway remains unclear.

(III) Interactions among the Modules. One crucial feature and contribution of the network is the inter-module interactions, revealed via the links among modules, that manifest the intricate relationship among biological pathways. Among the 13 modules in the network (Figure 3A), Modules 1, 3, 5 and 8 each interacts with another five, four, three and three modules, respectively, while Module 10 is involved in binary interactions with another two modules. For example, the modules of DNA replication, transcription, amino acid metabolism and protein biosynthesis (Modules 1, 3 and 10) interact as expected. However, from the interaction relationship between Modules 5 and 8 (Figure 3C), a novel pathway is discovered that explains the positive correlation between B_12_ and ethanol yield (Figure S5D): genes encoding ethanolamine and propanediol (the alternative energy sources for ethanol production) utilization proteins, acetate kinase (producing acetate and ATP) and alcohol dehydrogenase (the final step in ethanol fermentation) are all linked to the porphobilinogen deaminase (*teth5140318*; a key enzyme in B_12_ synthesis) and ATP-cobalamin adenosyltransferase (*teth5141943*; the enzyme converts vitamin B_12_ to coenzyme B_12_) (Figure 3C). Thus, via this pathway, B_12_ synthesis interacts with and promotes ethanol production. Therefore, the network reveals unrecognized pathway interactions and indentifies genes serving crucial junctions.

Additionally, the absence of inter-module interactions also contains crucial information. There are seven “standalone” modules in the network. For example, Module 8, Module 7 (mostly related to fructose utilization) and Module 4 (related to cellobiose utilization) are free of any connections or links, suggesting relative structural separation and functional independence among glucose, xylose, fructose and cellobiose catabolism in X514 (View II, Figure 2 and Figure S6; Part VII of Text S1).

Similarly, based on the number of links for each node, the network also reveals the relative importance of genes in biological processes [16]. Genes encoding the oligopeptide ABC transporter, xylose ABC transporter, PTS, binding protein-dependent transport systems, pyruvate oxidoreductase, acetate kinase and ethanolamine utilization protein are examples of genes with the highest number of connections (Table S11), suggesting active and important contributions by these genes to the glycobiome.

Furthermore, when integrated in an evolutionary perspective, the network reveals crucial metabolic and regulatory junctions. One prevalent feature of microbial genomes is the apparent redundancy of paralogs with individual contributions that can be elusive. One such example is the nine alcohol dehydrogenase (*adh*) genes in the X514 genome, which could play crucial roles in ethanologenesis by mediating the last and shared step of both pentose and hexose fermentations that converts aldehyde to ethanol. Two of them are NADPH-dependent *adh*s: *teth5140653* (*adhB*) and *teth5140654* (*adhA*); one of them is a bifunctional alcohol dehydrogenase/aldehyde dehydrogenase (*teth5140627*, *adhE*) [17], [18]. These three genes and four iron-containing *adh*s (*teth5140145/0241/0564/1935*) cluster in COG C, whereas an iron-containing *adh* (*teth5141808*) and a short-chain *adh* (*teth5141882*) cluster into COG Q (secondary metabolite catabolism) and COG E, respectively (Figure S7 and Table S12). The network reveals the various roles that these *adh*s play (Part VIII of Text S1). In particular, *teth1541935* is one of the most prominent (i.e., well-connected) nodes in the network. It is located in Module 8 (mostly xylose catabolism and energy production genes) and directly linked to 21 genes involved in ethanol conversion, particularly in B_12_-dependent ethanolamine utilization and propanediol utilization (Figure S5B). Moreover, *teth1541935* is induced under both fructose and xylose compared to glucose and is positively correlated with higher ethanol yields via the ethanolamine and propanediol pathways (Figure 3C).

The protein sequence-based phylogeny of all *adh*s in X514 and 39E (39E harbors seven *adh*s; Figure S7B) reveals that, except for *teth5141935* and *teth5140145* in X514 and *teth391597* in 39E, most X514 *adh*s have orthologs in 39E (a total of six such orthologous pairs) and are under stringent negative selection, suggesting a strong evolutionary pressure to preserve their functions (Figure S7B). *Teth5141935* and *0145* are the only two strain-specific *adh*s in X514, and the former coincides with the *teth5141935*, which prominently stands out in the network with 21 links (Figure S5B and Table S12; *teth5140145* is absent in the network). All of these data strongly identify *teth5141935* as a crucial junction in ethanol production and a key target in the rational perturbation of the network. Thus, of the nine *adh*s in the genome, seven are constitutively expressed, six are under negative selection (Ka/Ks <1), and two are X514 lineage-specific innovations with one of the two (*teth5141935*) serving as a crucial junction in energy production.

### Transcriptional choreographies of the *Thermoanaerobacter* glycobiome reveal the dynamics and the cooperating nature of pentose and hexose utilization

By aligning the three choreographies (glucose, xylose, and dual carbohydrate) progressing across the three growth phases, the network not only provides a “static” model for glycobiome structure and regulation, but reveals the dynamics of this process (View III in Figure 2).

Among the three choreographies, the differentially expressed genes (Figure S8) are mostly those in energy production (C), carbohydrate transport and metabolism (G), amino acid metabolism (E), coenzyme metabolism (H), and inorganic ion transport (P) (Figure S8C and S8D). However, there are a number of prominent discordances.

**Figure 4:**
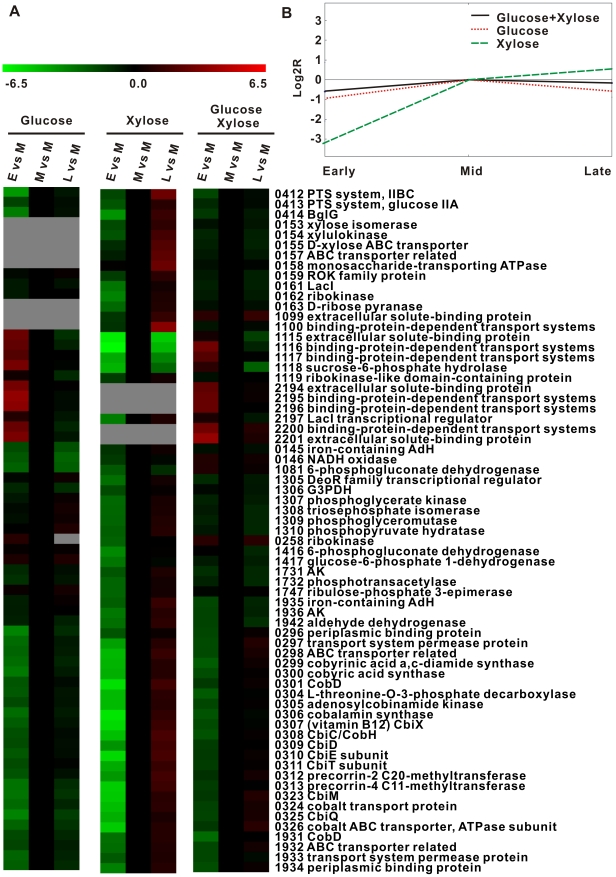
Dynamics of the *Thermoanaerobacter* glycobiome in the presence of glucose, xylose, or glucose-xylose. A) Expression ratios (log_2_ *R*) for representative genes involved in COG C, COG G, COG P and COG H across the time course are distinguished by colors: red, upregulated; black, no change; and green, downregulated. Those with a microarray signal-noise-ratio (SNR) <2 (i.e., expression level not quantifiable) are represented by the color gray. E: early exponential phase; M: mid exponential phase; and L: late exponential phase. B) Centroid graph of gene expression patterns during the exponential growth phase under glucose, xylose or glucose-xylose. Expression patterns are visualized with TM4 software [39].

First, glucose quickly switched on genes involved in carbon transport and metabolism (glycolysis and pentose phosphate pathway (PPP)) during early exponential phase, in both glucose alone and glucose-xylose growth conditions. In glucose alone, the two loci (*teth5141115-1118* and *teth5142194-2201*) encoding carbohydrate-binding proteins (and associated transporters in each locus) peaked at early exponential phase, whereas the glucose-specific IIA/IIBC of PTS (*teth5140412-0413*) peaked during mid exponential phase (Figure 4A). However, in the xylose-alone choreography, a relative delay (“xylose lag”) was observed: the xylose-specific binding protein genes (*teth5141099-1100*) peaked during mid exponential phase with the ABC transport systems (*teth5140986-0992* and *teth5140157-0158*) peaking during late exponential phase (Figure 4A). However, under the dual carbohydrate condition, the “xylose lag” disappeared. Indeed, genes encoding xylose- and glucose-binding proteins peaked during early exponential phase (except teth5141100), while carbohydrate transport systems of glucose-specific PTSs and xylose ABC transporters both peaked during mid exponential phase. For PPP and glycolysis pathway genes, this finding was consistent with that observed for transporter genes (Figure 4A). Thus, when both glucose and xylose are present, glucose apparently activates xylose transport and catabolism via monosaccharide binding proteins (during early exponential phase) and via monosaccharide transport, PPP and glycolysis genes (during mid exponential phase).

**Figure 5:**
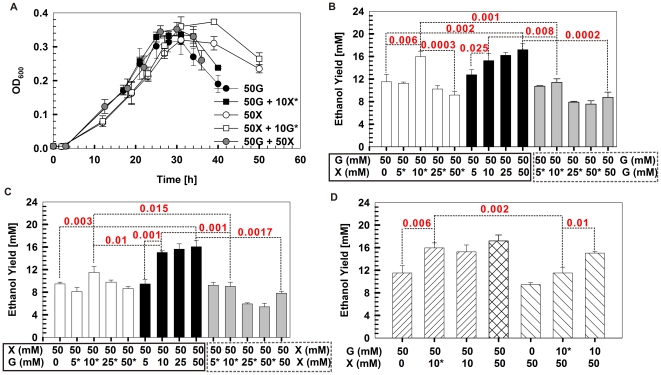
Ethanol yield enhancement via timing optimization and loading of specific carbon supplies. A) Growth curves of *Thermoanaerobacter* sp. X514 under different carbohydrates in defined medium. B, C and D) Ethanol yields under various timing and loading (and their combinations) conditions of specific carbohydrate substrates. Differences between groups were evaluated using one-tailed paired t-tests. For selected pairs that exhibited significant differences, p values are shown (those with p<0.05 are considered significant). *: the substrate (glucose or xylose) was introduced during mid exponential phase; G: glucose; and X: xylose.

Second, xylose extended cellular coenzyme activities and sustained cellular growth during late exponential phase, whether alone or as one of the dual substrates (Figure 1; also see Figure 5A). In each of the three choreographies, most genes involved in inorganic ion transport and coenzyme metabolism were poorly transcribed during early exponential phase but abundantly expressed during mid exponential phase (Figure 4A). However, their expression quickly decreased during late exponential phase under glucose but not under xylose or xylose-glucose. Examples of such genes include the putative cobalt transport (*teth5140323*-*0326*) and vitamin B_12_ coenzyme biosynthesis (*teth5140296*-*0321*) operons. The sustained high expression of these genes probably explains the prolonged stationary phase whenever xylose is present.

Therefore, the dual carbohydrate glycobiomes include the respective choreographic features of xylose- and glucose-alone glycobiomes (Figure 4B). In particular, glucose accelerated xylose utilization via activating xylose transport and catabolism genes, whereas xylose maintained and extended coenzyme activities and ion metabolism to delay cellular lysis (Figure 1A and Figure 5A). Such a structurally independent yet functionally collaborating interaction between pentose and hexose catabolism explains the robust glucose-xylose co-utilization and appears to be a key feature of the *Thermoanaerobacter* glycobiome.

### Collaborative pentose and hexose catabolism are exploited for product-yield via optimizing timing and loading of specific carbon supplies

To validate the network-derived findings on the distinct roles and cooperative nature of pentose and hexose, a series of batch fermentation experiments were devised in which, in a substrate-specific manner, the relative loading and timing (and their combinations) of the carbon supply were varied (Figure 5). Under dual carbohydrate conditions, the growth curve was similar to that of glucose alone, while the duration of the stationary phase was similar to that of xylose alone (Figure 5A). Moreover, when introducing xylose but not glucose into a glucose-alone culture, either at inoculation or at mid exponential phase, the stationary phase was extended (Figure 5A and Figure S9). Both findings are consistent with the hypothesized roles and cooperative nature of glucose and xylose.

Furthermore, the ethanol yields were also examined (Figure 5). First, the dual substrate condition yields significantly more ethanol than did either substrate alone. For example, glucose plus xylose (at 50 mM each) produced 89% more ethanol than did 100 mM glucose (*p* = 0.0002; Figure 5B) and 113% more ethanol than did 100 mM xylose (*p* = 0.0017; Figure 5C).

Second, under dual substrate, there is an additive, substrate concentration-dependent effect on the ethanol yield when supplementing one substrate for the other at inoculation. However, the effect disappeared beyond ∼10 mM of the supplementary substrate. Beyond this threshold, adding more of the supplementary substrate did not further improve ethanol yield. For example, on top of 50 mM glucose, 10 mM supplementary xylose yielded a level of ethanol similar to 50 mM supplementary xylose (*p* value not significant; Figure 5B). On top of 50 mM xylose, 10 mM supplementary glucose yielded a similar amount of ethanol to 50 mM supplementary glucose (*p* value not significant; Figure 5C).

Third, the timing of introducing the supplementary substrate is an important factor in determining ethanol yield. When supplementing 10 mM xylose with 50 mM glucose, no significant difference in ethanol yield was found whether introducing the supplementary substrate at inoculation or during mid exponential phase (Figure 5B) In fact, replacing the supplementary xylose with glucose lowered the ethanol yield (data not shown). However, adding 10 mM supplementary glucose at inoculation to 50 mM xylose yielded 31% more (*p* = 0.01) ethanol than adding the glucose during mid exponential phase (Figure 5C). Replacing supplementary glucose with xylose lowered the ethanol yield (data not shown). This finding is also consistent with the distinct yet collaborative action modes of glucose and xylose.

Finally, the substrate-specific and timing-dependent features were exploited to achieve optimal yield. For example, supplementing 10 mM xylose during mid exponential phase to 50 mM glucose (added at inoculation) yielded 39% more ethanol (*p* = 0.002) than did supplementing 10 mM glucose during mid exponential phase to 50-mM xylose (added at inoculation) (Figure 5D).

Therefore, the distinct yet collaborative glucose and xylose catabolism can be exploited to enhance ethanol yield via optimized timing and balanced loading of the carbon supply in a substrate-specific manner.

### Cellular model of the *Thermoanaerobacter* glycobiome

**Figure 6:**
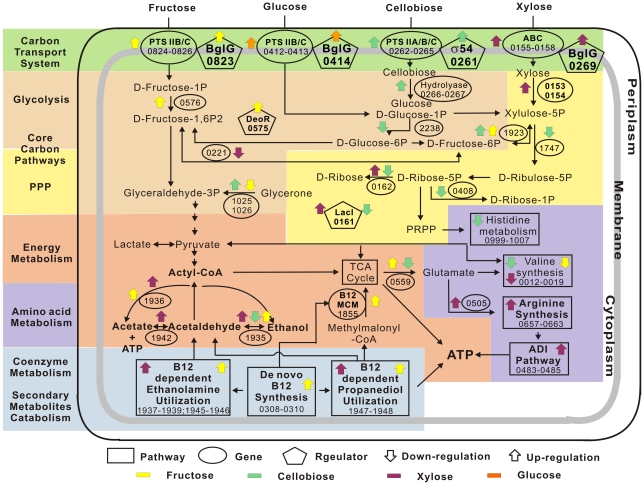
Cellular model of the *Thermoanaerobacter* glycobiome. Key transport, metabolic and regulatory genes and pathways are illustrated. Yellow arrows: genes specifically expressed under fructose (glucose as reference). Green arrows: those specifically expressed under cellobiose (glucose as reference). Purple arrows: those specifically expressed under xylose (glucose as reference). Orange arrows: the two putative operons specifically expressed under glucose but downregulated under all other substrates. Up arrow: upregulation; down arrow: downregulation. IDs of the corresponding genes in X514 are shown.

The structure and dynamics of the glycobiome enables the construction of a hypothetical cellular model of carbohydrate catabolism for thermophilic anaerobes (Figure 6), which highlights the features shared with and distinct from model microbes, such as *E. coli*. First, transcription factors, such as the BglG family anti-terminators, respond to carbon-substrate availability and activate both hexose-specific PTS transport systems and ABC pentose transport systems (in contrast to *E. coli* in which XylR regulates ABC xylose transport) [12]. Second, when the monosaccharides are transported into cells, another set of regulators is expressed: the DeoR-family regulator, responding to hexoses (e.g., fructose), activates glycolysis genes, while *lacI* and other members of the putative PPP operon that includes *lacI* are all induced under xylose (Figure 6). The two core pathways of carbon metabolism, glycolysis and PPP, consequently metabolize hexoses, pentoses and disaccharides. Third, the vitamin B_12_ pathway promotes ethanol production through B_12_-dependent ethanolamine and 1, 2-propanediol, while the ADI pathway revives the cell for sustained growth (Figure 6).

## Discussion

### Features of the network reconstruction approach

Our study demonstrates a strategy for the efficient and productive reconstruction of functional and regulatory networks based on a rational experimental design, a limited number of transcriptomes sampled and appropriate computational theories. First, to maximize the discriminating power of co-expression-based analysis, time courses (View III; Figure 2) were introduced in the experimental design in contrast to most previous studies in which only substrates (View I and II; Figure 2) were considered and only the mid exponential phase was sampled [19]. In this study, when only the 15 datasets from different substrates (15 microarrays) were used for network construction, no prominent biological insights were revealed (data not shown). Thus, the absence of the time course datasets would have prevented crucial insights, such as the nature of interactions between the glucose and xylose modules. Second, we demonstrated that valuable insights can emerge from a transcriptome dataset of a reasonable size (33 total microarrays; View I/II/III; Figure 2). Previous networks were usually constructed with thousands of microarrays [20], and the notion that the construction of biologically meaningful co-expression networks demands “scale” has largely limited such studies to only a few model organisms [21]. Third, one crucial factor determining network quality is the threshold of the Pearson correlation between genes. Methods for rationally choosing this threshold include those based on known biological information [22] and on the statistical comparison of randomized expression data [23], which usually are manual, subjective and require organisms with well-established biological backgrounds. Random matrix theory in an automatic and objective fashion distinguishes system-specific, non-random properties embedded in complex systems [10], [16], and thus is especially valuable in non-model organisms where little or no prior knowledge exits.

### Novel insights into thermoanaerobic carbohydrate catabolism

Hexose and pentose co-utilization is an extraordinary and industrially valued property among ethanologens. *Saccharomyces cerevisiae* and *Zymomonas mobilis* are not able to ferment pentose to ethanol [4], while *E. coli* and *B. subtilis* prefer hexoses over pentoses, due to CCR [6]. In *Thermoanaerobacter*, the intriguing trait of hexose and pentose co-utilization may be tentatively interpreted as an adaptation to barren environments where carbohydrates (and, in particular, monosaccharides, are scarce [8]). Simultaneous activation of catabolic pathways for all monosaccharides available may thus be viewed as an evolutionary acquisition with adaptive value. In addition, as an anaerobe, these cells lack an oxidative PPP for converting hexoses to pentoses [24]. Consequently, any preference for hexose would have compromised the many crucial metabolic processes where pentose played essential roles. Such environmental and evolutionary pressure left striking footprints on their regulatory mode as well. For example, in both *E. coli* and *B. subtilis*, the activation of xylose utilization genes uses XylR [12], [25], which activates xylose utilization loci. In *B. subtilis*, when glucose and xylose are both present, the transcriptional regulator catabolite control protein A (CcpA), which is activated by the hexose-induced phosphorylated histidine-containing protein (HPr), blocks XylR-binding sites upstream of xylose utilization loci, thus preventing xylose from being consumed until glucose is depleted. Alternatively, in *Thermoanaerobacter*, both the hexose- and pentose-transport systems appear to be regulated by BglGs, rather than by XylR for pentose. Our model suggests that one BglG (Teth5140269) positively regulates xylose ABC transporters (Teth5140157) under both xylose alone and dual carbon source (glucose and xylose), a second (Teth5140823) activates fructose-specific PTS transporters (Teth5140824-0826), and a third (Teth5140414) activates glucose-specific PTS transporters (Teth5140412-0413) (Figure 6 and Figure S3). This difference may contribute to the pentose-hexose co-utilization.

Compared to aerobes, *de novo* B_12_ synthesis is another advantageous trait in *Thermoanaerobacter*, a strict anaerobe, because B_12_ promotes ethanol production in the thermophilic anaerobe *C. thermocellum* [26]. Only strict anaerobes contain this trait because B_12_ is only anaerobically synthesized [27]. This trait simplifies nutrient requirements and promotes ethanol yields via ethanolamine and 1, 2-propanediol utilization pathways in X514, a clear advantage in ethanol production (Figure 3C). Furthermore, the ADI pathway, which is a survival strategy that delays host cell lysis and ethanol damage [15], functions only anaerobically. Under aerobic conditions, another arginine catabolism pathway (arginine to glutamate) is induced [28]. These features and their genetic basis distinguish the *Thermoanaerobacter* glycobiome and suggest novel exploitation strategies to enhance *Thermoanaerobacter* fermentation.

### Impact on CBP strain development for cost-effective cellulosic biofuel production

Mono- or co-cultures of cellulolytic and/or ethanologenic TGPAs (e.g., *C. thermocellum* and *T. ethanolicus* [2], [29]) are promising CBP organisms, but several challenges in their utilization remain. Our study reveals new strategies and targets for overcoming these hurdles.

First, glucose and xylose utilization can be enhanced by engineering specific transport apparatuses. At least five predicted operons, including both ABC transporters and PTSs, may be involved in transporting xylose or glucose, and their individual contributions can be elusive. Our results identify putative operons that are specifically affected by glucose (*teth5140412-0414*) and xylose (*teth5140153-0155* and *teth5140157-0158*), thereby presenting a prioritized list of engineering targets in X514 and related organisms. Second, cellobiose, which is abundant in lignocellosic hydrolysates, is a dimer of glucose. However, in X514, lower biomass and end products were produced under cellobiose than under glucose (Figure S1). The presence of Module 4 (related to cellobiose catabolism) in the network (Figure 3A) and the finding that these genes require a longer induction identify cellobiose utilization as one bottleneck in *Thermoanaerobacter* ethanol yield, reveal a relatively independent mechanism of cellobiose hydrolysis and transport and pinpoint *teth5140262-0267*, which encodes a disaccharide-specific transporter and glycoside hydrolyase cassette, as a key engineering target to improve cellobiose utilization. Third, one of the nine *adh*s in the genome, *teth5141935*, is identified as a top target for engineering ethanol yield. Adhs regulate the balance leading to ethanol; therefore, the perturbation of *teth5141935* should improve ethanol yield [30]. Because large arrays of paralogous *adh*s are the norm rather than the exception in microbes (9 in X514, 7 in *E. coli* and 7 in *S. cerevisiae*; [31]), our network-based approach for functionally distinguishing such paralogs should have broader applications. Finally, we uncovered the distinct properties of glucose and xylose as cellular fuels. Glucose and xylose modules overlap when both monosaccharides are present: the former activates xylose transport and catabolism during early exponential phase (via monosaccharide binding proteins) and mid exponential phase (via monosaccharide transport and PPP and glycolysis genes), while the latter delays cell lysis by sustaining coenzyme and ion metabolisms. This finding could be valuable to the design of more efficient carbon utilization devices and modules in synthetic biology. Furthermore, we experimentally demonstrated that these properties can be rationally exploited to enhance ethanol yield.

In summary, the newly discovered modular and precisely regulated network unveiled unique features of the *Thermoanaerobacter* glycobiome and revealed novel perturbation strategies and targets. Together with the genetic systems under development [30], [32], this network forms the foundation for rational engineering of both fermentation and cellular machineries in TGPA and other ethanologens for efficient hexose-pentose co-utilization. Furthermore, we demonstrated a strategy for cost-effective network reconstruction. The expanded applications of such a strategy and approaches in monocultures (e.g., exploring network fluidity or evolution) and mixed communities could provide new insights into interacting pathways, cells and cellular populations.

## Materials and Methods

### Oligonucleotide probe design and microarray construction

Microarrays for X514 were constructed with 70-mer nucleotide probes. This microarray includes 2315 probes covering 2322 annotated gene sequences. Among these probes, 20 probes were designed from 20 ORFs randomly selected from the human genome and were used as negative controls. Of the 2365 gene sequences used for probe design, no probes could be designed for 43 of them, which were thus not covered by the microarray. Each probe was replicated twice on the microarray, resulting in two sub-arrays on the slide. Gene-specific and group-specific oligonucleotide probes were designed with CommOligo 2.0 software [33] based on the following criteria: identity ≤85%, stretch ≤20 bases, and free energy ≥−40 kCal/Mol to non-target sequences for gene-specific probes; and identity ≥96%, stretch ≥55 bases, and free energy ≤−90 kCal/Mol to target sequences for group-specific probes. All designed oligonucleotides were commercially synthesized by MWG Biotech Inc. (High Point, NC) and prepared, as described by Li et al. [33]. Detailed information of the probes and their targets were reported in Table S13.

### Cell growth conditions and product profiling

*Thermoanaerobacter* sp. X514 was grown anaerobically in defined medium [8] supplemented with either 50 mM glucose, xylose, fructose or cellobiose as the sole carbon source or 50 mM glucose plus 50 mM xylose as dual carbohydrates at 60°C without shaking. X514 was passaged five times on the substrate of interest in defined medium before inoculation. Each sample was harvested during the early exponential (OD_600_∼0.1), mid exponential (OD_600_∼0.17) and late exponential (OD_600_∼0.27) phases. Next, cell pellets were frozen immediately in liquid N_2_ and stored at −80°C prior to RNA extraction. All of the samples were prepared and analyzed in triplicate.

Concentrations of acetate, ethanol and lactate were analyzed using a high-performance liquid chromatograph (Agilent Technologies, CA) equipped with a variable wavelength (190–600 nm) detector (VWD, with UV absorption at 245 nm) and an ion exclusion column (Aminex HPX-87H, 300 mm×7.8 mm, Bio-Rad Laboratories, CA) at a column temperature of 55°C. The mobile phase consisted of 0.025% sulfuric acid at a flow rate of 0.6 ml/min. An Aminex HPX-87P column (Bio-Rad) was used with deionized water as the mobile phase to measure the sugar concentrations.

### Acquisition and analysis of global gene-expression profiles

Total cellular RNA and genomic DNA were isolated and labeled, as previously described [34], [35]. The Cy3-labeled genomic DNA was used as a common reference to co-hybridize the Cy5-labeled RNA samples on each slide. Hybridization was performed using a TECAN HS4800 Pro Hybridization Station (Tecan, NC) by following the manufacturer's instructions. After hybridization, slides were scanned using a ProScanArray microarray analysis system (Perkin Elmer, MA).

To determine the signal intensity of fluorescence for each spot, the scanned 16-bit TIFF images were analyzed by ImaGene 6.1 software (Biodiscovery Inc., EI Segundo, CA) as described by [34]. Details of the microarray data analysis were previously described [34]. Typically, a cutoff |log_2_ *R*| ≥1.0 and |Z score| ≥2.0 were used to determine changes of significance. The complete microarray dataset was deposited as NCBI GEO GSE24458.

### Validation of microarray-based gene-expression profiles: real-time quantification PCR (qRT-PCR)

The robustness of the high-density oligonucleotide microarray-based expression profiles was tested by qRT-PCR of a selected set of 20 genes distributed in different putative operons (Table S14). The specific primer pair for each gene and their respective sequences were listed in Table S14. The qRT-PCR analysis was performed according to a previously described protocol [34]. Copy numbers of the target gene transcripts were determined via comparison with standard curves, and log_2_ *R* ratios were subsequently determined. A high correlation coefficient of 0.932 was observed between the qRT-PCR and the microarray results (Figure S10), validating the reproducibility of the microarray data.

### Construction of the gene co-expression network

All 33 microarray datasets from the sole and dual carbon sources were used for the construction of a gene co-expression network based on random matrix theory [16]. For each spot on the microarray, a normalized Cy5/Cy3 ratio was calculated, and then logarithmic transformation of the ratio was performed. The network was generated with a Pearson correlation coefficient cut-off at 0.94 between each pair of genes [16]. The modules were separated by fast greedy modularity optimization [36]. This algorithm divides an entirely unclustered network where each node in the graph forms a singleton community into organized modular structures by computing modularity between two communities based on the connections between nodes until a maximum modularity value is reached [37].

### Phylogenetic analysis of orthologous and paralogous genes

The amino acid sequences of the nine and seven *adh*s of X514 and 39E, respectively, were extracted from the NCBI reference genome sequences NC_010320.1 and NC_010321.1. These *adh* sequences were analyzed by ClustalW based on MEGA 4 software. Ka/Ks was calculated by the PAML software. Ka/Ks <1 suggests negative selection while Ka/Ks>1 indicates positive selection [38].

## Supporting Information

Figure S1 Thermoanaerobic Carbohydrate Fermentation by X514. A) Sugar utilization. B) Acetate, lactate and ethanol production. All experiments were performed in triplicate, and standard deviations are shown. Differences were evaluated by one-tailed paired t-tests, with p<0.05 considered significant. C) Time courses of ethanol production (early, mid and late exponential phase). D) Time courses of sugar utilization (square) and growth curves (circle) under glucose (black) and xylose (white). E) Growth curves under different mono-carbohydrates during exponential growth phase. Data are fitted with the *Y = ae* *^b^* *X* equation at the exponential growth phase.
(TIF)

Figure S2 One Sub-Module with Newly Revealed Functions and Module 4 (mostly related to cellobiose utilization or encoding hypothetical proteins). A) The genes in the sub-module are the first neighbors of *teth5141834-1835*. B) Module 4. Blue lines indicate positive correlation coefficients. The color code is as in Figure 3A.
(TIF)

Figure S3 Sub-modules Encoding Novel Regulatory Functions. Genes in these sub-modules are the first neighbors of *teth5140269* (A), *teth5140159* (B), *teth5141590* (C) and *teth5141567*, *teth5141275* and *teth5141161* (D). Color code is as in Figure 3A. Blue lines indicate positive correlation coefficients.
(TIF)

Figure S4 Organization of the *xyl* Loci of *Escherichia coli*, *Thermoanaerobacter ethanolicus* 39E and *Thermoanaerobacter* sp. X514. IDs of the corresponding *xyl* genes in X514 are also shown.
(TIF)

Figure S5 Selected Modules and Sub-Modules in the *Thermoanaerobacter* Glycobiome Network. A) Module 7 (mostly fructose catabolism genes). B) Sub-module involving the *adh* (*teth5141935*). C) Two sub-modules involving the *adh*s (*teth5141982* and *teth5140241*). Genes in these sub-modules are the first neighbors of *teth5141935*, *1882* and *0241*. Color code is as in Figure 3A. Blue lines indicate positive correlation coefficients. D) A novel pathway identified in this study underlies the positive correlation between B_12_ and ethanol yield. IDs of the corresponding genes in X514 are also shown.
(TIF)

Figure S6 Genome-Wide Expression Pattern of *Thermoanaerobacter* sp. X514 under Glucose-Xylose. The genes and predicted operons under COG C and G clusters are shown, which are involved in glucose and xylose transport and catabolism. Xyl or X: xylose; and Glu or G: glucose.
(TIF)

Figure S7 Transcriptional Features and Evolutionary Origins of Alcohol Dehydrogenase Genes. A) Transcriptional programs of the genes. G: glucose; X: xylose; F: fructose; C: cellobiose; E: early exponential phase; M: mid exponential phase; and L: late exponential phase. B) Phylogenetic tree of the *adh*s in X514 and 39E. Numbers below the branches are bootstrap (500 times) values, while those above indicate Ka/Ks.
(TIF)

Figure S8 Statistical Analysis of the Genes Differentially Expressed in Different Growth Conditions. A) Numbers of the up- or downregulated genes under each condition. Each column represents the number of genes with significant expression changes (|log_2_ *R*| ≥1 and |Z score| ≥2) under the corresponding sugars: fructose (F), glucose (G), xylose (X) or cellobiose (C), early exponential phase of glucose (eG), mid exponential phase of glucose (mG), late exponential phase of glucose (lG), early exponential phase of xylose (eX), mid exponential phase of xylose (mX) and late exponential phase of xylose (lX). B) Differentially expressed genes (over the total number of significantly changed genes) in each COG category, under mono-carbohydrate cultures at mid exponential phase. C) Differentially expressed genes (over the total number of significantly changed genes) at different growth phases under glucose alone. D) Differentially expressed genes (over the total number of significantly changed genes) at different growth phases under xylose alone. Asterisks indicate that the majority of differentially expressed genes are in these COG categories.
(TIF)

Figure S9 Growth Curves of *Thermoanaerobacter* sp. X514 with 10 mM Glucose or Xylose Introduced at either Inoculation or Mid Exponential Phase. The supplementary substrate was respectively added to 50 mM xylose or glucose added at inoculation. *: the substrate (glucose or xylose) was introduced at mid exponential phase. G: glucose; and X: xylose.
(TIF)

Figure S10 Real-time Quantitative RT-PCR (qRT-PCR) Analysis of Selected Genes for Validating Microarray Data. The induction levels were compared among 14 genes induced by xylose, 14 genes induced by glucose, and 12 genes related to mid exponential phase growth of X514. All of the genes were randomly selected. The comparison was plotted on log_2_ *R*, which was determined by microarrays (*x-*axis) and qRT-PCR (*y-*axis).
(TIF)

Table S1 The 614 Genes Constituting the Reconstructed Genome-Wide Carbon Utilization Network of *Thermoanaerobacter* sp. X514.
(XLS)

Table S2 Up- or Downregulated “Hypothetical Genes” of *Thermoanaerobacter* sp. X514 under the Different Carbohydrates. Bold fonts indicate |Z score| ≥2.
(DOC)

Table S3 Up- or Downregulated Genes in the Transportation and Metabolism of Carbohydrates (COG G) in *Thermoanaerobacter* sp. X514 under Xylose or Glucose-Xylose. Bold fonts indicate |Z score| ≥2. Glu: glucose; and Xyl: xylose.
(DOC)

Table S4 Up- or Downregulated Genes in Energy Metabolism (COG C) in *Thermoanaerobacter* sp. X514 under Xylose or Glucose-Xylose. Bold fonts indicate |Z score| ≥2. Glu: glucose; and Xyl: xylose.
(DOC)

Table S5 Up- or Downregulated Genes in Amino Acid Metabolism (COG E) in *Thermoanaerobacter* sp. X514 under Xylose or Glucose-Xylose. Bold fonts indicate |Z score| ≥2. Glu: glucose; and Xyl: xylose.
(DOC)

Table S6 Up- or Downregulated Genes in the Transportation and Metabolism of Carbohydrates (COG G) in *Thermoanaerobacter* sp. X514 under Fructose. Bold fonts indicate |Z score| ≥2.
(DOC)

Table S7 Up- or Downregulated Genes in Amino Acid Metabolism (COG E) in *Thermoanaerobacter* sp. X514 under Fructose. Bold fonts indicate |Z score| ≥ 2.
(DOC)

Table S8 Up- or Downregulated Genes in Energy Metabolism (COG C) in *Thermoanaerobacter* sp. X514 under Fructose. Bold fonts indicate |Z score| ≥2.
(DOC)

Table S9 Up- or Downregulated Genes in the Transportation and Metabolism of Carbohydrates (COG G) in *Thermoanaerobacter* sp. X514 under Cellobiose. Bold fonts indicate |Z score| ≥2.
(DOC)

Table S10 Up- or Downregulated Genes in Energy Metabolism (COG C) in *Thermoanaerobacter* sp. X514 under Cellobiose. Bold fonts indicate |Z score| ≥2.
(DOC)

Table S11 Top 23 Genes with the Highest Numbers of Connections in the *Thermoanaerobacter* Glycobiome Network.
(DOC)

Table S12 Expression of the Nine Alcohol Dehydrogenase Genes from X514 under Different Carbohydrates. Bold fonts indicate |Z score| ≥2. G: glucose; X: xylose; F: fructose; C: cellobiose; Early: early exponential phase; Mid: mid exponential phase; and Late: late exponential phase.
(DOC)

Table S13 Complete List of Probes and Genes on the *Thermoanaerobacter* sp. X514 Whole-Genome Microarray for Gene Expression Profiling.
(XLS)

Table S14 Primers and Sequences Used for qRT-PCR.
(XLS)

Text S1 Supplemental Materials.
(DOC)
