# Supplementary figures and images for: The Thermoanaerobacter Glycobiome Reveals Mechanisms of Pentose and Hexose Co-Utilization in Bacteria
Source: PLoS Genet. 2011 Oct 13;7(10):e1002318. doi: 10.1371/journal.pgen.1002318 (PMC3192829; doi:10.1371/journal.pgen.1002318)

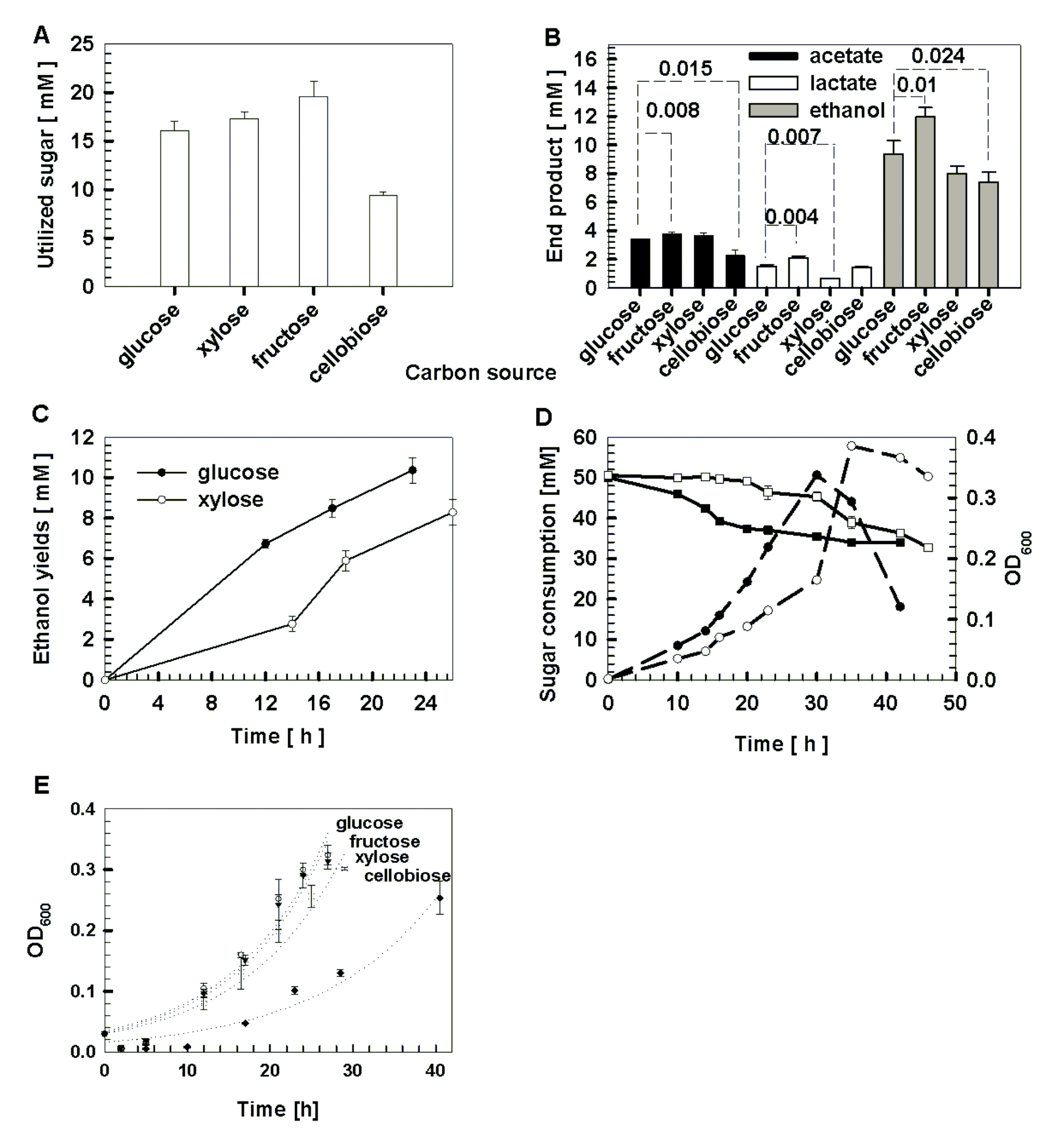

Supplement: Figure S1 — Thermoanaerobic Carbohydrate Fermentation by X514. A) Sugar utilization. B) Acetate, lactate and ethanol production. All experiments were performed in triplicate, and standard deviations are shown. Differences were evaluated by one-tailed paired t-tests, with p<0.05 considered significant. C) Time courses of ethanol production (early, mid and late exponential phase). D) Time courses of sugar utilization (square) and growth curves (circle) under glucose (black) and xylose (white). E) Growth curves under different mono-carbohydrates during exponential growth phase. Data are fitted with the Y = ae b X equation at the exponential growth phase. (TIF) [file pgen.1002318.s001.tif]

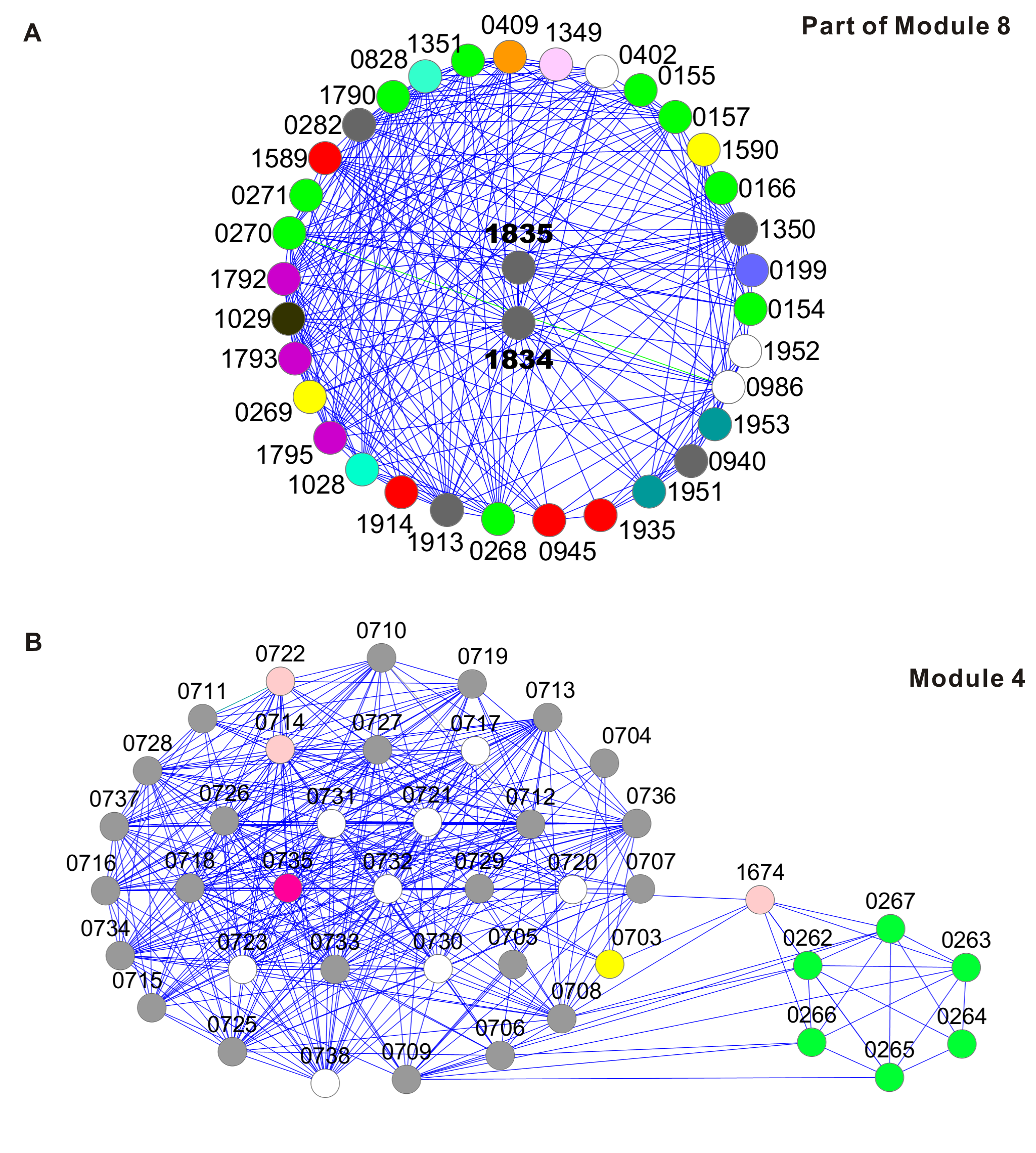

Supplement: Figure S2 — One Sub-Module with Newly Revealed Functions and Module 4 (mostly related to cellobiose utilization or encoding hypothetical proteins). A) The genes in the sub-module are the first neighbors of teth5141834-1835. B) Module 4. Blue lines indicate positive correlation coefficients. The color code is as in Figure 3A. (TIF) [file pgen.1002318.s002.tif]

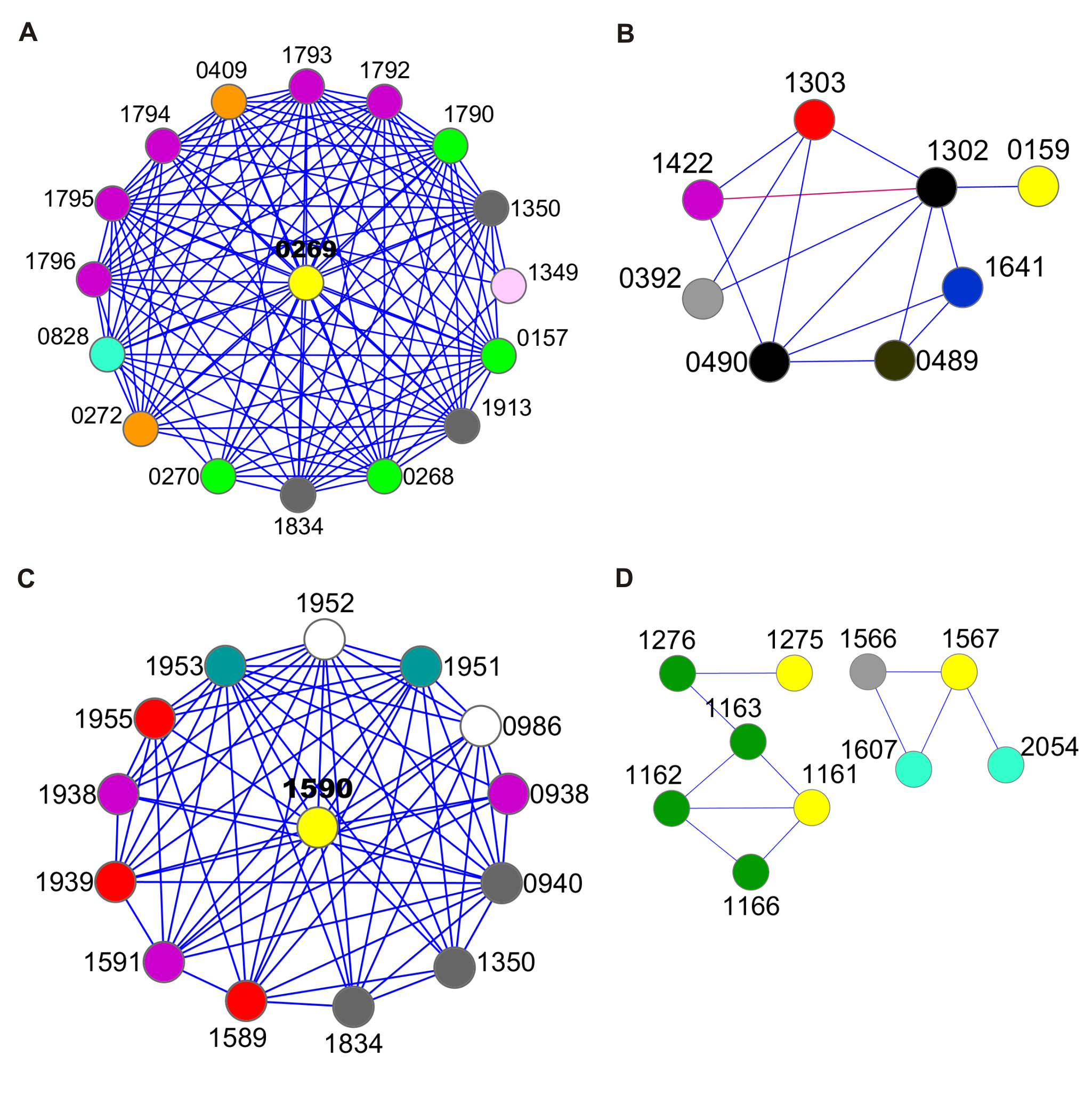

Supplement: Figure S3 — Sub-modules Encoding Novel Regulatory Functions. Genes in these sub-modules are the first neighbors of teth5140269 (A), teth5140159 (B), teth5141590 (C) and teth5141567, teth5141275 and teth5141161 (D). Color code is as in Figure 3A. Blue lines indicate positive correlation coefficients. (TIF) [file pgen.1002318.s003.tif]

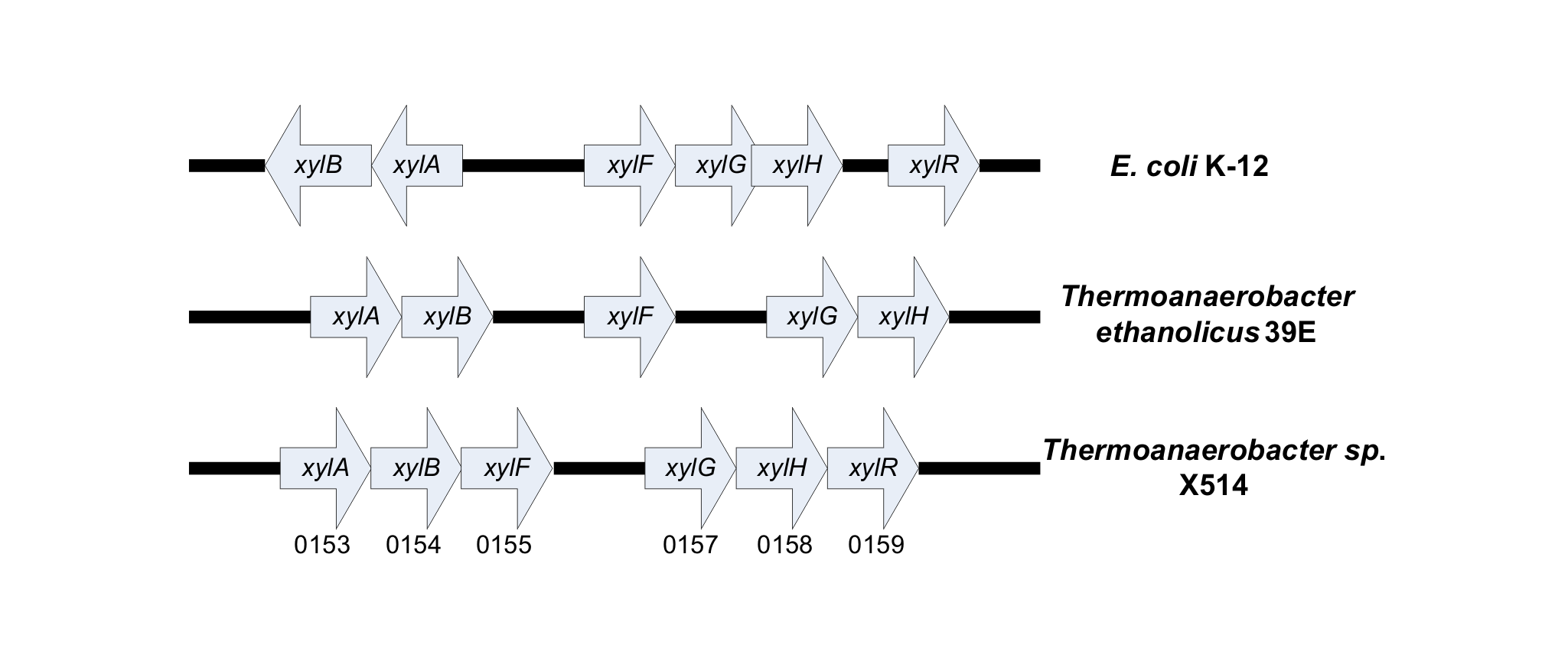

Supplement: Figure S4 — Organization of the xyl Loci of Escherichia coli, Thermoanaerobacter ethanolicus 39E and Thermoanaerobacter sp. X514. IDs of the corresponding xyl genes in X514 are also shown. (TIF) [file pgen.1002318.s004.tif]

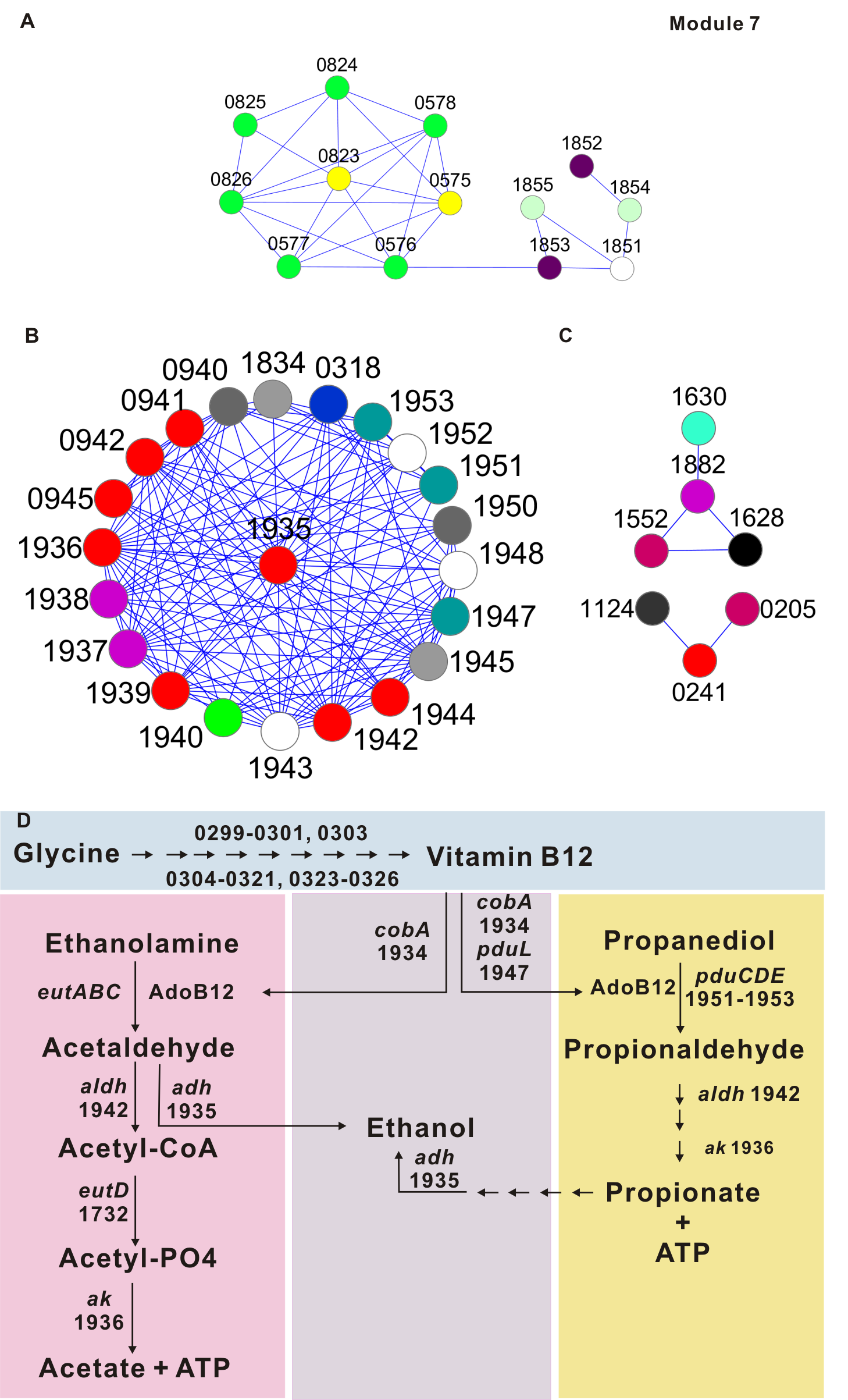

Supplement: Figure S5 — Selected Modules and Sub-Modules in the Thermoanaerobacter Glycobiome Network. A) Module 7 (mostly fructose catabolism genes). B) Sub-module involving the adh (teth5141935). C) Two sub-modules involving the adhs (teth5141982 and teth5140241). Genes in these sub-modules are the first neighbors of teth5141935, 1882 and 0241. Color code is as in Figure 3A. Blue lines indicate positive correlation coefficients. D) A novel pathway identified in this study underlies the positive correlation between B12 and ethanol yield. IDs of the corresponding genes in X514 are also shown. (TIF) [file pgen.1002318.s005.tif]

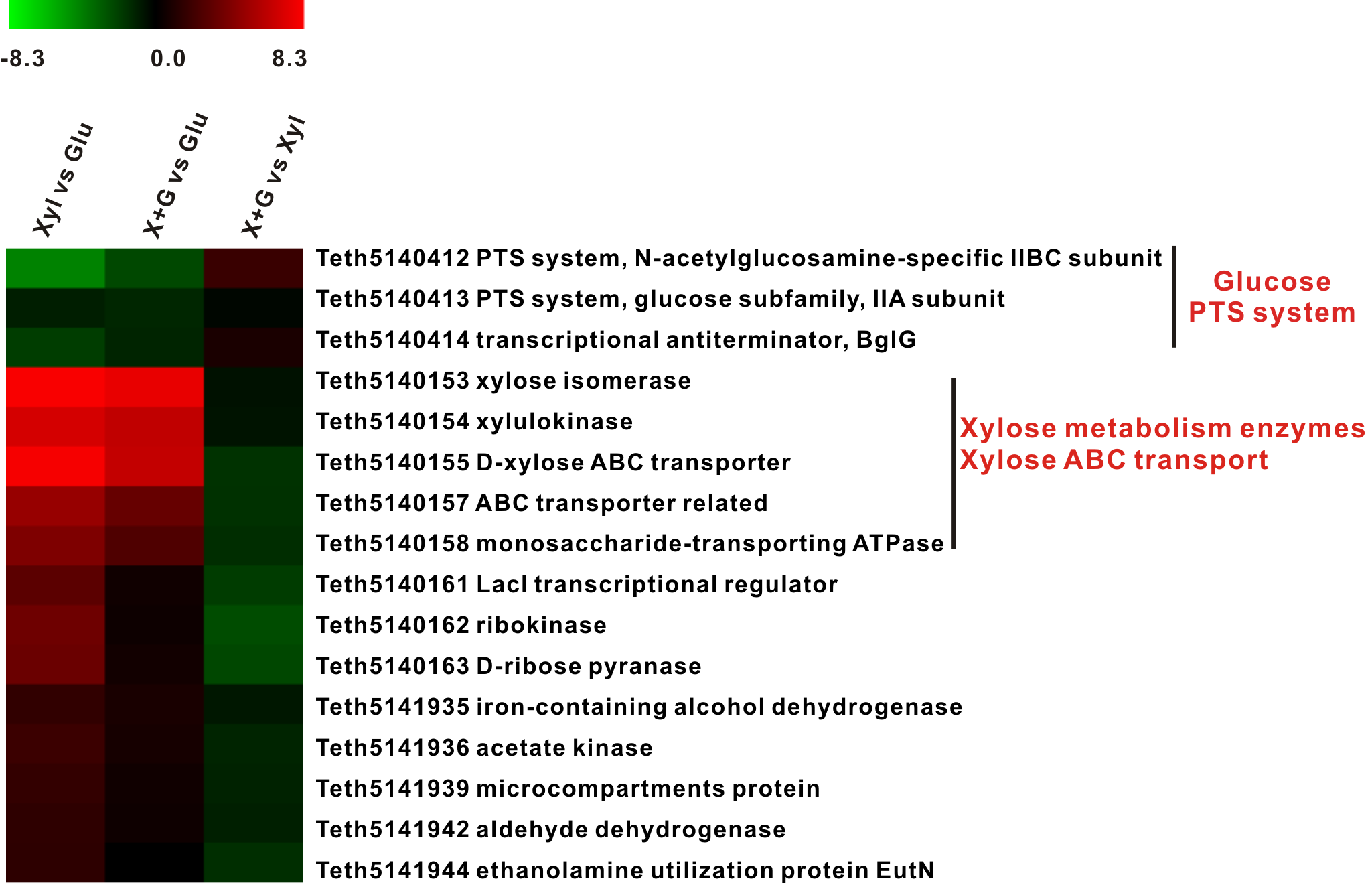

Supplement: Figure S6 — Genome-Wide Expression Pattern of Thermoanaerobacter sp. X514 under Glucose-Xylose. The genes and predicted operons under COG C and G clusters are shown, which are involved in glucose and xylose transport and catabolism. Xyl or X: xylose; and Glu or G: glucose. (TIF) [file pgen.1002318.s006.tif]

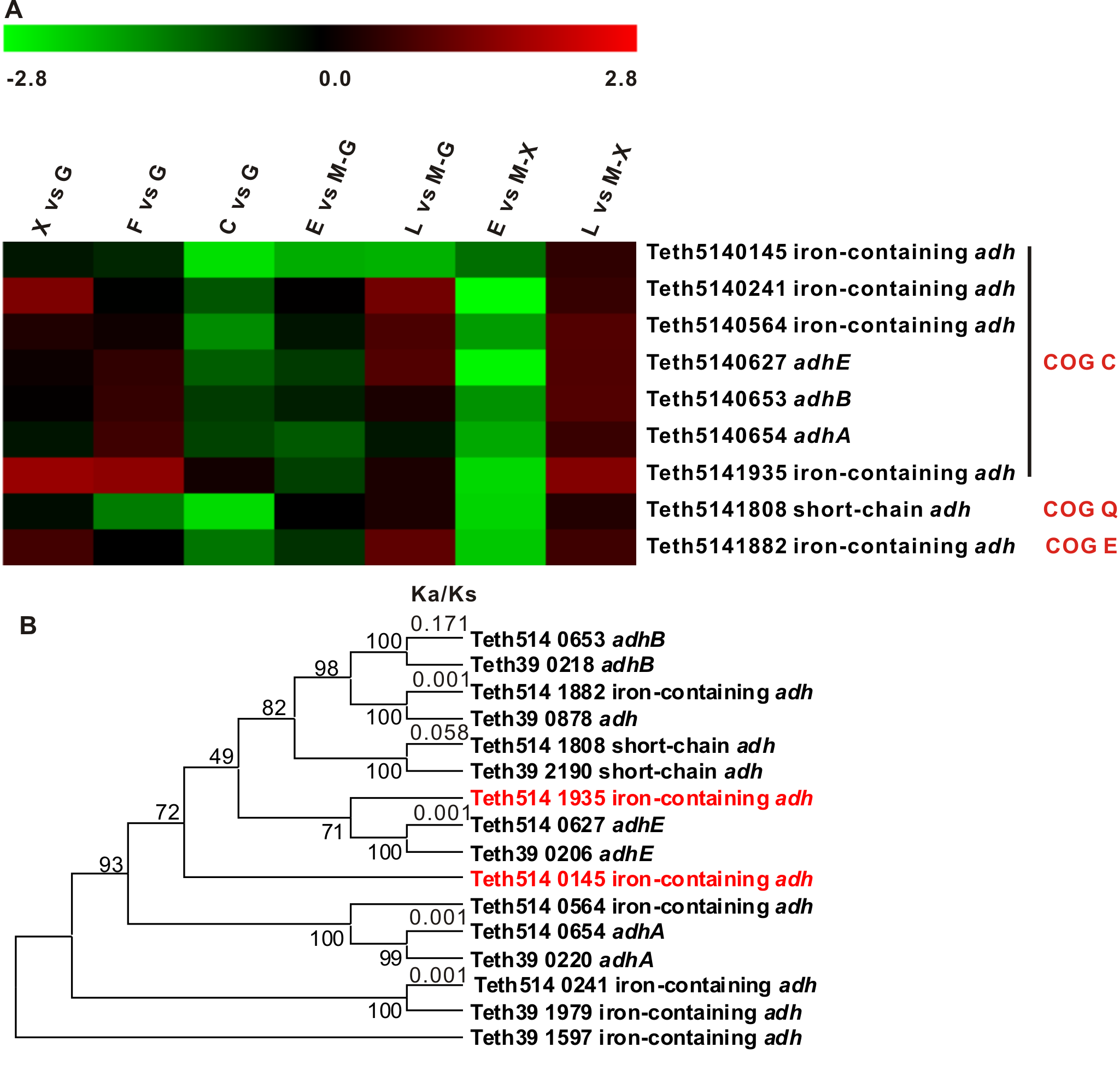

Supplement: Figure S7 — Transcriptional Features and Evolutionary Origins of Alcohol Dehydrogenase Genes. A) Transcriptional programs of the genes. G: glucose; X: xylose; F: fructose; C: cellobiose; E: early exponential phase; M: mid exponential phase; and L: late exponential phase. B) Phylogenetic tree of the adhs in X514 and 39E. Numbers below the branches are bootstrap (500 times) values, while those above indicate Ka/Ks. (TIF) [file pgen.1002318.s007.tif]

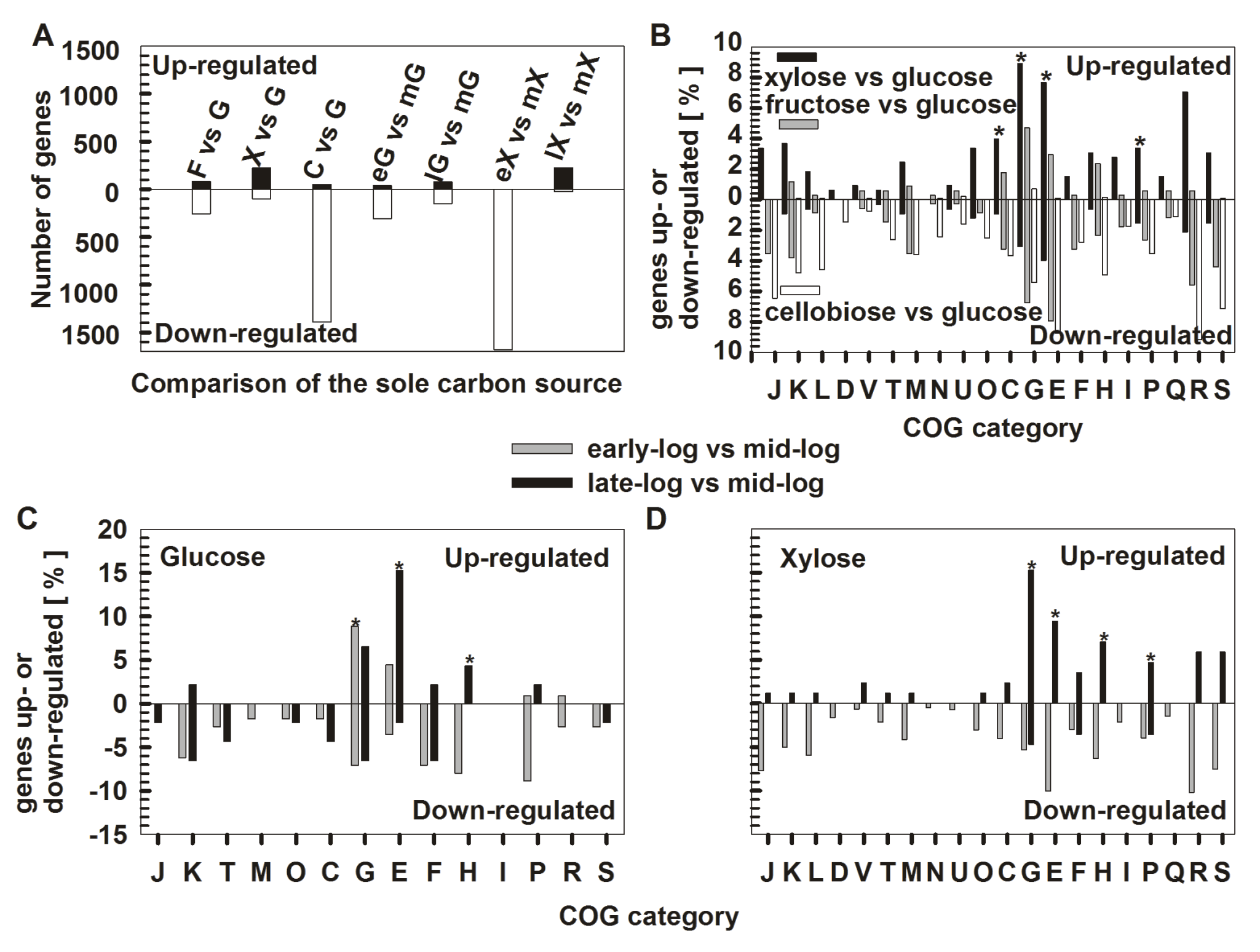

Supplement: Figure S8 — Statistical Analysis of the Genes Differentially Expressed in Different Growth Conditions. A) Numbers of the up- or downregulated genes under each condition. Each column represents the number of genes with significant expression changes (|log2 R| ≥1 and |Z score| ≥2) under the corresponding sugars: fructose (F), glucose (G), xylose (X) or cellobiose (C), early exponential phase of glucose (eG), mid exponential phase of glucose (mG), late exponential phase of glucose (lG), early exponential phase of xylose (eX), mid exponential phase of xylose (mX) and late exponential phase of xylose (lX). B) Differentially expressed genes (over the total number of significantly changed genes) in each COG category, under mono-carbohydrate cultures at mid exponential phase. C) Differentially expressed genes (over the total number of significantly changed genes) at different growth phases under glucose alone. D) Differentially expressed genes (over the total number of significantly changed genes) at different growth phases under xylose alone. Asterisks indicate that the majority of differentially expressed genes are in these COG categories. (TIF) [file pgen.1002318.s008.tif]

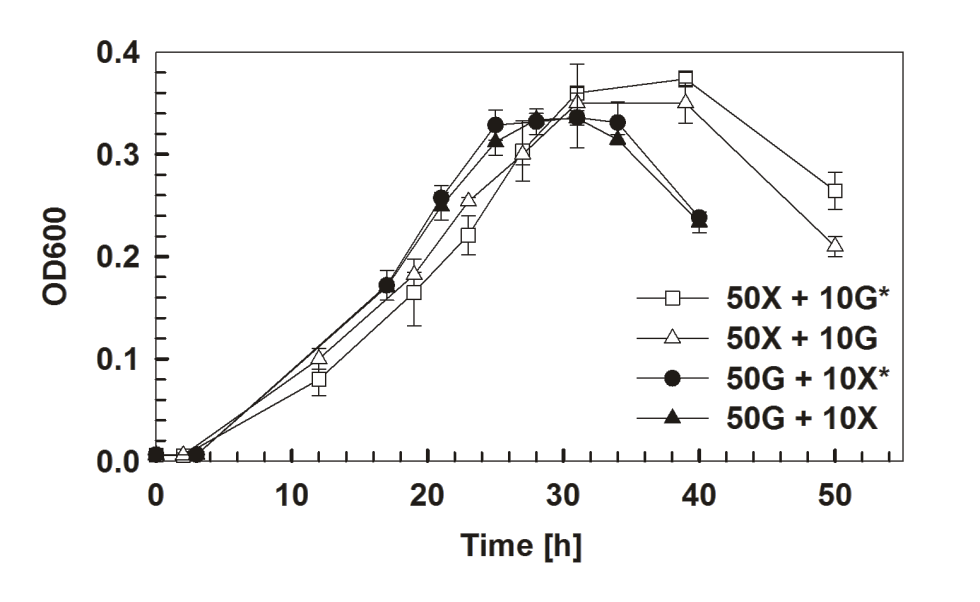

Supplement: Figure S9 — Growth Curves of Thermoanaerobacter sp. X514 with 10 mM Glucose or Xylose Introduced at either Inoculation or Mid Exponential Phase. The supplementary substrate was respectively added to 50 mM xylose or glucose added at inoculation. *: the substrate (glucose or xylose) was introduced at mid exponential phase. G: glucose; and X: xylose. (TIF) [file pgen.1002318.s009.tif]

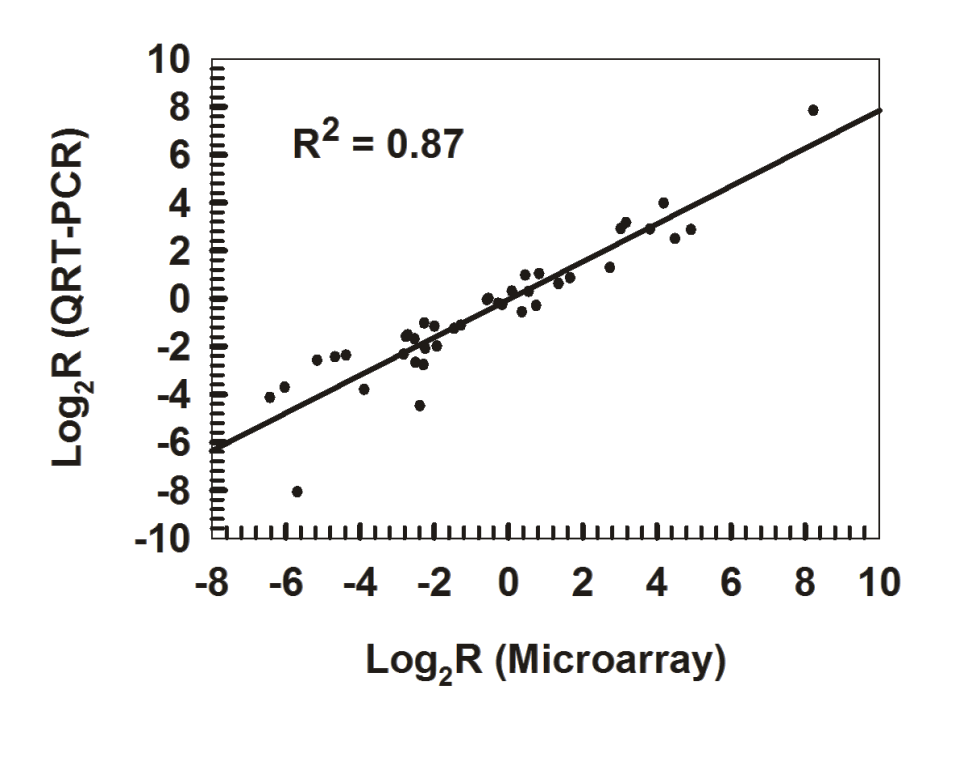

Supplement: Figure S10 — Real-time Quantitative RT-PCR (qRT-PCR) Analysis of Selected Genes for Validating Microarray Data. The induction levels were compared among 14 genes induced by xylose, 14 genes induced by glucose, and 12 genes related to mid exponential phase growth of X514. All of the genes were randomly selected. The comparison was plotted on log2 R, which was determined by microarrays (x-axis) and qRT-PCR (y-axis). (TIF) [file pgen.1002318.s010.tif]
