# Supplementary material for: The Thermoanaerobacter Glycobiome Reveals Mechanisms of Pentose and Hexose Co-Utilization in Bacteria
Source: PLoS Genet. 2011 Oct 13;7(10):e1002318. doi: 10.1371/journal.pgen.1002318 (PMC3192829; doi:10.1371/journal.pgen.1002318)
Supplement: Table S2 — Up- or Downregulated “Hypothetical Genes” of Thermoanaerobacter sp. X514 under the Different Carbohydrates. Bold fonts indicate |Z score| ≥2. (DOC) [file pgen.1002318.s012.doc]

**Table S2. Up- or Down-regulated “Hypothetical Genes” of *Thermoanaerobacter* sp. X514 under the Different Carbohydrates. Bold fonts indicated |Z score|≥ 2.**

| **Gene ID** | **No. of links** | **Annotation** | **COG** | **Cellobiose vs Glucose** | | **Xylose vs Glucose** | | **Frcutose vs Glucose** | |
| --- | --- | --- | --- | --- | --- | --- | --- | --- | --- |
|  |  |  |  | **log_2_*R*** | **Z score** | **log_2_*R*** | **Z score** | **log_2_*R*** | **Z score** |
| Teth5140703 | 7 | XRE family transcriptional regulator | K | 4.71 | **3.23** | -1.48 | **-2.34** | 1.15 | 1.66 |
| Teth5140704 | 5 | hypothetical protein |  | 4.32 | **2.54** | -1.51 | **-2.37** | 0.98 | 1.51 |
| Teth5140705 | 2 | hypothetical protein |  | 4.70 | **3.70** | -1.48 | **-2.73** | 0.64 | 0.98 |
| Teth5140706 | 12 | hypothetical protein |  | 4.15 | **3.88** | -2.06 | **-3.79** | 1.95 | **2.56** |
| Teth5140707 | 11 | hypothetical protein |  | 3.86 | **3.64** | -1.92 | **-3.48** | 1.82 | **2.36** |
| Teth5140708 | 23 | hypothetical protein |  | 4.49 | **7.32** | -0.93 | -1.83 | 1.74 | **3.08** |
| Teth5140709 | 19 | hypothetical protein |  | 3.02 | 1.62 | -0.97 | -1.45 | 1.02 | 1.08 |
| Teth5140710 | 18 | hypothetical protein |  | 3.12 | **3.26** | -2.41 | **-4.12** | 1.50 | 1.33 |
| Teth5140711 | 13 | hypothetical protein |  | 2.19 | **2.92** | -2.45 | **-4.38** | 1.04 | 1.15 |
| Teth5140712 | 28 | hypothetical protein |  | 3.32 | **5.58** | -2.15 | **-4.14** | 1.59 | **2.09** |
| Teth5140713 | 25 | hypothetical protein |  | 3.59 | **3.97** | -2.06 | **-3.86** | 2.19 | **2.19** |
| Teth5140714 | 25 | Type IV secretory pathway VirB4 components-like protein | U | 3.79 | **6.16** | -1.76 | **-3.38** | 2.57 | **3.46** |
| Teth5140715 | 18 | hypothetical protein |  | 3.41 | **4.66** | -2.62 | **-4.98** | 2.39 | **2.93** |
| Teth5140716 | 22 | hypothetical protein |  | 3.17 | **6.00** | -3.21 | **-6.38** | 1.74 | **2.99** |
| Teth5140717 | 17 | peptidase M23B |  | 3.83 | **5.73** | -1.65 | **-3.17** | 2.72 | **3.91** |
| Teth5140718 | 30 | hypothetical protein |  | 3.18 | **5.50** | -1.46 | **-2.86** | 1.39 | **2.31** |
| Teth5140719 | 19 | hypothetical protein |  | 3.23 | **5.58** | -1.08 | **-2.09** | 1.42 | **2.26** |
| Teth5140720 | 16 | SAF domain-containing protein |  | 3.54 | **5.77** | -1.19 | **-2.30** | 1.17 | 1.96 |
| Teth5141945 | 18 | hypothetical protein |  | NA | NA | 1.41 | **2.53** | 1.54 | **2.72** |
| Teth5141946 | 3 | ethanolamine utilization protein EutJ family protein | E | NA | NA | 0.97 | 1.57 | 1.99 | **2.79** |
