# Supplementary material for: The Thermoanaerobacter Glycobiome Reveals Mechanisms of Pentose and Hexose Co-Utilization in Bacteria
Source: PLoS Genet. 2011 Oct 13;7(10):e1002318. doi: 10.1371/journal.pgen.1002318 (PMC3192829; doi:10.1371/journal.pgen.1002318)
Supplement: Table S3 — Up- or Downregulated Genes in the Transportation and Metabolism of Carbohydrates (COG G) in Thermoanaerobacter sp. X514 under Xylose or Glucose-Xylose. Bold fonts indicate |Z score| ≥2. Glu: glucose; and Xyl: xylose. (DOC) [file pgen.1002318.s013.doc]

**Table S3. Up- or Down-regulated Genes in the Transportation and Metabolism of Carbohydrates (COG G) for *Thermoanaerobacter* sp. X514 under Xylose or Glucose-Xylose. Bold fonts indicated |Z score|≥ 2. Glu: glucose, Xyl: xylose**

| **Gene ID** | **Annotation** | **Xyl vs Glu** | | **Glu+Xyl vs Glu** | | **Glu + Xyl vs Xyl** | |
| --- | --- | --- | --- | --- | --- | --- | --- |
| **A. Carbon transport** | | **log_2_*R*** | **Z score** | **log_2_*R*** | **Z score** | **log_2_*R*** | **Z score** |
| Teth5140157 | ABC transporter related, XylG | 5.00 | **9.36** | 3.40 | **5.83** | -1.60 | **-2.59** |
| Teth5140158 | monosaccharide-transporting ATPase, XylH | 4.18 | **7.90** | 2.62 | **5.13** | -1.47 | **-2.68** |
| Teth5140159 | ROK family protein, XylR | 0.92 | 1.29 | 0.26 | 0.35 | -0.57 | -0.72 |
| Teth5140160 | oxidoreductase domain-containing protein | 0.66 | 0.70 | 0.46 | 0.44 | 0.08 | 0.07 |
| Teth5140226 | ABC-type xylose transport system periplasmic component-like protein | -4.66 | **-8.74** | -6.58 | **-12.06** | -1.89 | **-3.75** |
| Teth5140227 | ABC transporter related | -3.19 | **-6.24** | -4.89 | **-9.51** | -1.51 | **-2.98** |
| Teth5140412 | PTS system, N-acetylglucosamine-specific IIBC subunit | -4.25 | **-7.23** | -2.35 | **-3.60** | 1.92 | **3.41** |
| Teth5140413 | PTS system, glucose subfamily, IIA subunit | -1.00 | **-1.93** | -1.24 | **-2.31** | -0.22 | -0.42 |
| Teth5140414 | transcriptional antiterminator, BglG | -1.99 | **-3.74** | -1.18 | **-2.15** | 0.91 | 1.69 |
| Teth5140168 | RpiR family transcriptional regulator | 0.32 | 0.57 | 0.00 | 0.00 | -0.20 | -0.32 |
| Teth5140169 | PTS system, glucose subfamily, IIA subunit | 0.09 | 0.18 | -0.73 | -1.44 | -0.82 | -1.62 |
| Teth5140170 | phosphotransferase system, EIIC | -0.27 | -0.53 | -1.63 | **-3.18** | -1.34 | **-2.64** |
| Teth5141115 | extracellular solute-binding protein | 4.53 | **9.01** | 1.59 | **3.08** | -2.94 | **-5.76** |
| Teth5141116 | binding-protein-dependent transport systems inner membrane component | 4.74 | **9.44** | -0.10 | -0.20 | -4.84 | **-9.59** |
| Teth5141117 | binding-protein-dependent transport systems inner membrane component | 3.28 | **6.53** | -0.81 | -1.59 | -4.09 | **-8.09** |
| Teth5141118 | sucrose-6-phosphate hydrolase | 3.33 | **6.61** | 1.25 | **2.46** | -2.08 | **-4.13** |
| Teth5141181 | extracellular solute-binding protein | 0.13 | 0.24 | 0.17 | 0.28 | 0.04 | 0.07 |
| Teth5141182 | binding-protein-dependent transport systems inner membrane component | -0.09 | -0.17 | -1.05 | **-2.04** | -0.91 | -1.76 |
| Teth5141183 | binding-protein-dependent transport systems inner membrane component | -0.05 | -0.09 | -0.41 | -0.72 | -0.32 | -0.58 |
| **B. Carbon metabolism** | |  |  |  |  |  |  |
| Teth5140153 | xylose isomerase, XylA | 8.22 | **10.90** | 7.65 | **7.18** | -0.56 | -0.44 |
| Teth5140154 | Xylulokinase, XylB | 7.03 | **10.05** | 6.35 | **7.11** | -0.65 | -0.61 |
| Teth5140155 | D-xylose ABC transporter, periplasmic substrate-binding protein, XylF | 8.11 | **12.52** | 6.45 | **9.65** | -1.65 | **-2.00** |
| Teth5140161 | periplasmic binding protein/LacI transcriptional regulator | 3.03 | **4.53** | 0.56 | 1.08 | -1.97 | **-2.70** |
| Teth5140162 | ribokinase | 3.67 | **4.77** | 0.46 | 0.86 | -2.48 | **-2.83** |
| Teth5140163 | D-ribose pyranase | 3.54 | **5.93** | 0.63 | 1.22 | -2.33 | **-3.66** |
| Teth5140164 | ABC transporter related | 3.38 | **5.00** | -0.05 | -0.09 | -2.79 | **-3.74** |
| Teth5140165 | monosaccharide-transporting ATPase | 2.78 | **4.39** | -0.54 | -1.04 | -2.67 | **-3.95** |
| Teth5140166 | monosaccharide-transporting ATPase | 0.42 | 0.75 | -1.03 | -1.97 | -1.19 | **-2.04** |
| Teth5140971 | 6-phosphofructokinase | -2.04 | **-2.18** | -2.18 | **-2.16** | 0.32 | 0.39 |
| Teth5140221 | fructose 1 6-bisphosphatase-like protein | -2.74 | **-4.76** | -2.53 | **-4.11** | 0.22 | 0.43 |
