# Supplementary material for: The Thermoanaerobacter Glycobiome Reveals Mechanisms of Pentose and Hexose Co-Utilization in Bacteria
Source: PLoS Genet. 2011 Oct 13;7(10):e1002318. doi: 10.1371/journal.pgen.1002318 (PMC3192829; doi:10.1371/journal.pgen.1002318)
Supplement: Table S4 — Up- or Downregulated Genes in Energy Metabolism (COG C) in Thermoanaerobacter sp. X514 under Xylose or Glucose-Xylose. Bold fonts indicate |Z score| ≥2. Glu: glucose; and Xyl: xylose. (DOC) [file pgen.1002318.s014.doc]

**Table S4. Up- or Down-regulated Genes in Energy Metabolism (COG C) for *Thermoanaerobacter* sp. X514 under Xylose or Glucose-Xylose. Bold fonts indicated |Z score|≥ 2. Glu: glucose, Xyl: xylose**

| **Gene ID** | **Annotation** | **Xyl vs Glu** | | **Glu+Xyl vs Glu** | | **Glu + Xyl vs Xyl** | |
| --- | --- | --- | --- | --- | --- | --- | --- |
|  |  | **log_2_*R*** | **Z score** | **log_2_*R*** | **Z score** | **log_2_*R*** | **Z score** |
| Teth5140415 | trans-homoaconitate synthase | -1.76 | **-3.49** | -2.94 | **-5.82** | -1.04 | **-2.04** |
| Teth5140416 | aconitate hydratase | -1.87 | **-3.68** | -2.73 | **-5.31** | -0.73 | -1.42 |
| Teth5141935 | iron-containing alcohol dehydrogenase | 1.66 | **2.64** | 0.86 | 1.25 | -0.76 | -1.20 |
| Teth5141936 | acetate kinase | 1.97 | **3.34** | 0.78 | 1.20 | -1.19 | **-2.00** |
| Teth5141939 | microcompartments protein | 1.72 | **3.06** | 0.57 | 0.96 | -1.13 | **-2.03** |
| Teth5141942 | aldehyde dehydrogenase | 1.53 | **2.05** | 0.51 | 0.57 | -1.02 | -1.53 |
| Teth5141944 | ethanolamine utilization protein EutN/carboxysome structural protein Ccml | 1.50 | **2.23** | -0.02 | -0.03 | -1.51 | **-2.30** |
| Teth5141981 | NAD(P)H-dependent glycerol-3-phosphate dehydrogenase | 1.43 | **2.30** | 1.03 | 1.56 | -0.29 | -0.45 |
| Teth5141404 | radical SAM domain-containing protein | 1.25 | **2.26** | 0.98 | 1.75 | -0.10 | -0.17 |
| Teth5141405 | glycerophosphodiester phosphodiesterase | 1.31 | **2.47** | 0.68 | 1.31 | -0.46 | -0.83 |
| Teth5140017 | 3-isopropylmalate dehydrogenase | -2.31 | **-4.57** | -1.55 | **-3.04** | 1.02 | **2.01** |
| Teth5141303 | acylphosphatase | 1.89 | **2.60** | 0.64 | 0.71 | -0.82 | -0.97 |
| Teth5140145 | iron-containing alcohol dehydrogenase | -0.33 | -0.63 | -1.01 | -1.92 | -0.64 | -1.27 |
| Teth5140146 | NADH:flavin oxidoreductase/NADH oxidase | -0.87 | -1.66 | -2.13 | **-3.92** | -1.25 | **-2.47** |
| Teth5141378 | NADH:flavin oxidoreductase/NADH oxidase | 0.31 | 0.60 | -0.88 | -1.62 | -1.18 | **-2.20** |
