# Supplementary material for: The Thermoanaerobacter Glycobiome Reveals Mechanisms of Pentose and Hexose Co-Utilization in Bacteria
Source: PLoS Genet. 2011 Oct 13;7(10):e1002318. doi: 10.1371/journal.pgen.1002318 (PMC3192829; doi:10.1371/journal.pgen.1002318)
Supplement: Table S5 — Up- or Downregulated Genes in Amino Acid Metabolism (COG E) in Thermoanaerobacter sp. X514 under Xylose or Glucose-Xylose. Bold fonts indicate |Z score| ≥2. Glu: glucose; and Xyl: xylose. (DOC) [file pgen.1002318.s015.doc]

**Table S5. Up- or Down-regulated Genes in Amino Acid Metabolism (COG E) for *Thermoanaerobacter* sp. X514 under Xylose or Glucose-Xylose. Bold fonts indicated |Z score|≥ 2. Glu: glucose, Xyl: xylose**

| **Gene ID** | **Annotation** | **Xyl vs Glu** | | **Glu+Xyl vs Glu** | | **Glu + Xyl vs Xyl** | |
| --- | --- | --- | --- | --- | --- | --- | --- |
| **A. Amino acid metabolism** | | **log_2_*R*** | **Z score** | **log_2_*R*** | **Z score** | **log_2_*R*** | **Z score** |
| Teth5140657 | N-acetyl-gamma-glutamyl-phosphate reductase | 4.41 | **3.68** | 3.87 | **2.72** | -0.28 | -0.22 |
| Teth5140658 | bifunctional ornithine acetyltransferase/N-acetylglutamate synthase protein | 3.97 | **3.15** | 3.58 | **2.41** | 0.12 | 0.09 |
| Teth5140659 | acetylglutamate kinase | 3.30 | **2.86** | 2.47 | 1.95 | 0.11 | 0.09 |
| Teth5140660 | acetylornithine and succinylornithine aminotransferase | 3.00 | **2.87** | 2.62 | **2.31** | 0.83 | 0.79 |
| Teth5140663 | argininosuccinate synthase | 2.03 | **2.32** | 1.47 | 1.64 | 0.92 | 1.07 |
| Teth5140505 | ferredoxin-dependent glutamate synthase | 1.44 | **2.15** | 1.41 | 1.90 | 0.03 | 0.05 |
| Teth5141937 | ethanolamine utilization protein-like protein | 1.72 | **2.73** | 0.77 | 1.14 | -0.92 | -1.44 |
| Teth5141938 | microcompartments protein | 1.80 | **3.06** | 0.76 | 1.22 | -1.02 | -1.70 |
| Teth5140012 | acetolactate synthase, small subunit | -1.39 | **-2.66** | -1.23 | **-2.24** | 0.17 | 0.32 |
| Teth5140013 | ketol-acid reductoisomerase | -1.46 | **-2.91** | -1.14 | **-2.25** | 0.31 | 0.62 |
| Teth5140014 | pyruvate carboxyltransferase | -1.85 | **-3.56** | -2.18 | **-4.10** | -0.24 | -0.47 |
| Teth5140015 | 3-isopropylmalate dehydratase large subunit | -1.18 | **-2.30** | -1.26 | **-2.38** | 0.03 | 0.06 |
| Teth5140017 | 3-isopropylmalate dehydrogenase (Valine, leucine and isoleucine biosynthesis) | -2.31 | **-4.57** | -1.55 | **-3.04** | 1.02 | **2.01** |
| Teth5140018 | dihydroxy-acid dehydratase (Valine, leucine and isoleucine biosynthesis) | -1.62 | **-3.17** | -2.35 | **-4.54** | -0.63 | -1.24 |
| Teth5140483 | arginine deiminase | 0.04 | 0.08 | -2.49 | **-4.88** | -2.49 | **-4.90** |
| Teth5140484 | ornithine carbamoyltransferase | 0.43 | 0.68 | -0.04 | -0.07 | -0.35 | -0.53 |
| Teth5140485 | carbamate kinase | 0.24 | 0.43 | -0.04 | -0.06 | -0.20 | -0.33 |
| Teth5142306 | D-3-phosphoglycerate dehydrogenase | 1.62 | 1.87 | 2.16 | **2.31** | 0.81 | 0.82 |
| Teth5142307 | alanine--glyoxylate transaminase | 1.58 | **2.30** | 2.09 | **2.88** | 0.65 | 0.88 |
| Teth5142309 | homoserine kinase | 0.83 | 1.43 | 1.85 | **2.72** | 1.09 | 1.68 |
| Teth5142310 | threonine synthase | 0.90 | 1.63 | 1.31 | **2.19** | 0.58 | 0.97 |
| Teth5142311 | homoserine dehydrogenase | 1.17 | **2.04** | 2.24 | **3.20** | 1.13 | 1.65 |
| **B. Amino acid transport** | |  |  |  |  |  |  |
| Teth5140468 | extracellular solute-binding protein | 1.30 | **2.17** | 0.17 | 0.27 | -1.08 | -1.87 |
| Teth5140469 | polar amino acid ABC transporter, inner membrane subunit | 1.23 | **2.18** | 0.45 | 0.82 | -0.49 | -0.82 |
| Teth5140470 | ABC transporter related | 1.42 | **2.06** | 0.79 | 1.28 | -0.14 | -0.19 |
| Teth5141793 | oligopeptide/dipeptide ABC transporter, ATPase subunit | 1.01 | 1.97 | 1.01 | 1.97 | -1.59 | **-3.08** |
| Teth5141794 | binding-protein-dependent transport systems inner membrane component | 0.87 | 1.65 | -0.50 | -0.94 | -1.33 | **-2.53** |
| Teth5141795 | binding-protein-dependent transport systems inner membrane component | 0.99 | 1.94 | -0.39 | -0.75 | -1.37 | **-2.68** |
| Teth5141796 | extracellular solute-binding protein | 1.07 | **2.07** | -0.34 | -0.66 | -1.39 | **-2.65** |
