# Supplementary material for: The Thermoanaerobacter Glycobiome Reveals Mechanisms of Pentose and Hexose Co-Utilization in Bacteria
Source: PLoS Genet. 2011 Oct 13;7(10):e1002318. doi: 10.1371/journal.pgen.1002318 (PMC3192829; doi:10.1371/journal.pgen.1002318)
Supplement: Table S6 — Up- or Downregulated Genes in the Transportation and Metabolism of Carbohydrates (COG G) in Thermoanaerobacter sp. X514 under Fructose. Bold fonts indicate |Z score| ≥2. (DOC) [file pgen.1002318.s016.doc]

**Table S6. Up- or Down-regulated Genes in the Transportation and Metabolism of Carbohydrates (COG G) for *Thermoanaerobacter* sp. X514 under Fructose.** Bold fonts indicated |Z score|≥ 2.

| **Gene ID** | **Annotation** | **Fructose vs Glucose** | |
| --- | --- | --- | --- |
| **A. Carbon transport** | | **log_2_*R*** | **Z score** |
| Teth5140823 | transcriptional antiterminator, BglG | 3.77 | **6.67** |
| Teth5140824 | putative PTS IIA-like nitrogen-regulatory protein PtsN | 4.20 | **7.79** |
| Teth5140825 | PTS system, fructose subfamily, IIC subunit | 6.05 | **9.34** |
| Teth5140826 | PTS system, fructose-specific, IIB subunnit | 3.28 | **6.33** |
| Teth5140577 | putative PTS IIA-like nitrogen-regulatory protein PtsN | 4.78 | **9.21** |
| Teth5140578 | PTS system, fructose subfamily, IIC subunit | 4.95 | **8.56** |
| Teth5140132 | PTS system fructose subfamily IIA component | -3.01 | **-6.01** |
| Teth5140134 | PTS system mannose/fructose/sorbose family IID component | -1.84 | **-3.67** |
| Teth5140412 | PTS system, N-acetylglucosamine-specific IIBC subunit | -5.69 | **-9.61** |
| Teth5140413 | PTS system, glucose subfamily, IIA subunit | -2.15 | **-4.19** |
| Teth5140414 | transcriptional antiterminator, BglG | -4.06 | **-7.73** |
| Teth5140168 | RpiR family transcriptional regulator | -0.71 | -1.35 |
| Teth5140169 | PTS system, glucose subfamily, IIA subunit | -1.31 | **-2.61** |
| Teth5140170 | phosphotransferase system, EIIC | -0.54 | -1.06 |
| Teth5140171 | hypothetical protein | -0.60 | -1.17 |
| **B. Carbon metabolism** | |  |  |
| Teth5141081 | 6-phosphogluconate dehydrogenase | -1.38 | **-2.23** |
| Teth5140575 | DeoR family transcriptional regulator | 4.84 | **9.17** |
| Teth5140576 | 1-phosphofructokinase | 5.96 | **11.75** |
| Teth5141923 | transketolase domain-containing protein | 1.35 | **2.69** |
| Teth5141025 | dihydroxyacetone kinase, DhaK subunit | -1.85 | **-3.63** |
| Teth5141026 | dihydroxyacetone kinase, L subunit | -1.35 | **-2.55** |
