# Supplementary material for: The Thermoanaerobacter Glycobiome Reveals Mechanisms of Pentose and Hexose Co-Utilization in Bacteria
Source: PLoS Genet. 2011 Oct 13;7(10):e1002318. doi: 10.1371/journal.pgen.1002318 (PMC3192829; doi:10.1371/journal.pgen.1002318)
Supplement: Table S7 — Up- or Downregulated Genes in Amino Acid Metabolism (COG E) in Thermoanaerobacter sp. X514 under Fructose. Bold fonts indicate |Z score| ≥ 2. (DOC) [file pgen.1002318.s017.doc]

**Table S7. Up- or Down-regulated Genes in Amino Acid Metabolism (COG E) for *Thermoanaerobacter* sp. X514 under Fructose.** Bold fonts indicated |Z score|≥ 2.

| **Gene ID** | **Annotation** | **Fructose vs Glucose** | |
| --- | --- | --- | --- |
| **A. Amino acid metabolism** | | **log_2_*R*** | **Z score** |
| Teth5140013 | ketol-acid reductoisomerase | -1.15 | **-2.28** |
| Teth5140014 | pyruvate carboxyltransferase | -1.19 | **-2.27** |
| Teth5140015 | 3-isopropylmalate dehydratase large subunit | 2.71 | **3.55** |
| Teth5140017 | 3-isopropylmalate dehydrogenase | -1.06 | **-2.09** |
| Teth5140661 | carbamoyl phosphate synthase small subunit | -3.97 | **-6.89** |
| Teth5140662 | carbamoyl-phosphate synthase, large subunit | -3.45 | **-3.87** |
| Teth5142106 | asparagine synthetase B | -2.09 | **-4.00** |
| Teth5141337 | asparaginase/glutaminase | -1.63 | **-3.22** |
| Teth5142348 | peptidase M1, membrane alanine aminopeptidase | -1.59 | **-3.11** |
| Teth5141204 | putative alpha-isopropylmalate/homocitrate synthase family transferase | -1.95 | **-3.87** |
| Teth5141937 | ethanolamine utilization protein-like protein | 1.60 | **2.51** |
| Teth5141938 | microcompartments protein | 1.29 | **2.25** |
| Teth5141939 | microcompartments protein | 1.30 | **2.34** |
| Teth5141945 | hypothetical protein | 1.54 | **2.72** |
| Teth5141946 | ethanolamine utilization protein EutJ family protein | 1.99 | **2.79** |
| Teth5141947 | propanediol utilization protein | 1.79 | **2.19** |
| Teth5141948 | microcompartments protein | 1.39 | **2.33** |
| Teth5141853 | LAO/AO transport system ATPase | 1.39 | **2.56** |
| Teth5141855 | methylmalonyl-CoA mutase, large subunit | 1.46 | **2.57** |
| Teth5142307 | alanine--glyoxylate transaminase | 1.49 | **2.34** |
| **B. Amino acid transport** | |  |  |
| Teth5141201 | glycine betaine/L-proline ABC transporter, ATPase subunit | -1.94 | **-3.72** |
| Teth5141202 | binding-protein-dependent transport systems inner membrane component | -1.04 | **-2.02** |
| Teth5141853 | LAO/AO transport system ATPase | 1.39 | **2.56** |
