# Supplementary material for: The Thermoanaerobacter Glycobiome Reveals Mechanisms of Pentose and Hexose Co-Utilization in Bacteria
Source: PLoS Genet. 2011 Oct 13;7(10):e1002318. doi: 10.1371/journal.pgen.1002318 (PMC3192829; doi:10.1371/journal.pgen.1002318)
Supplement: Table S8 — Up- or Downregulated Genes in Energy Metabolism (COG C) in Thermoanaerobacter sp. X514 under Fructose. Bold fonts indicate |Z score| ≥2. (DOC) [file pgen.1002318.s018.doc]

**Table S8. Up- or Down-regulated Genes in Energy Metabolism (COG C) for *Thermoanaerobacter* sp. X514 under Fructose.** Bold fonts indicated |Z score|≥ 2.

| **Gene ID** | **Annotation** | **Fructose vs Glucose** | |
| --- | --- | --- | --- |
|  |  | **log_2_*R*** | **Z score** |
| Teth5140218 | dehydrogenase (flavoprotein)-like protein | -2.72 | **-5.25** |
| Teth5140219 | dehydrogenase (flavoprotein)-like protein | -1.86 | **-3.62** |
| Teth5140415 | trans-homoaconitate synthase | -2.41 | **-4.81** |
| Teth5140416 | aconitate hydratase | -2.18 | **-4.32** |
| Teth5140830 | malate dehydrogenase | -1.51 | **-2.97** |
| Teth5141935 | iron-containing alcohol dehydrogenase | 1.54 | **2.56** |
| Teth5141936 | acetate kinase | 1.56 | **2.60** |
| Teth5140502 | ferredoxin-NADP(+) reductase subunit alpha | 1.45 | **2.57** |
| Teth5140560 | 4Fe-4S ferredoxin iron-sulfur binding domain-containing protein | 1.56 | **3.08** |
| Teth5141939 | microcompartments protein | 1.30 | **2.34** |
