# Supplementary material for: The Thermoanaerobacter Glycobiome Reveals Mechanisms of Pentose and Hexose Co-Utilization in Bacteria
Source: PLoS Genet. 2011 Oct 13;7(10):e1002318. doi: 10.1371/journal.pgen.1002318 (PMC3192829; doi:10.1371/journal.pgen.1002318)
Supplement: Table S9 — Up- or Downregulated Genes in the Transportation and Metabolism of Carbohydrates (COG G) in Thermoanaerobacter sp. X514 under Cellobiose. Bold fonts indicate |Z score| ≥2. (DOC) [file pgen.1002318.s019.doc]

**Table S9. Up- or Down-regulated Genes in the Transportation and Metabolism of Carbohydrates (COG G) for *Thermoanaerobacter* sp. X514 under Cellobiose.** Bold fonts indicated |Z score|≥ 2.

| **Gene ID** | **Annotation** | | **Cellobiose vs Glucose** | |
| --- | --- | --- | --- | --- |
| **A. Carbon transport** | | | **log_2_*R*** | **Z score** |
| Teth5140412 | | PTS system, N-acetylglucosamine-specific IIBC subunit | -7.10 | **-11.77** |
| Teth5140413 | | PTS system, glucose subfamily, IIA subunit | -2.74 | **-5.33** |
| Teth5140414 | | transcriptional antiterminator, BglG | -5.28 | **-9.95** |
| Teth5140168 | | RpiR family transcriptional regulator | -1.95 | **-3.66** |
| Teth5140169 | | PTS system, glucose subfamily, IIA subunit | -2.12 | **-4.24** |
| Teth5140170 | | phosphotransferase system, EIIC | -2.90 | **-5.69** |
| Teth5140171 | | hypothetical protein | -1.22 | **-2.38** |
| Teth5140262 | | PTS system lactose/cellobiose family IIC subunit | 4.72 | **7.21** |
| Teth5140263 | | YdjC family protein | 5.12 | **7.92** |
| Teth5140264 | | phosphotransferase system, lactose/cellobiose-specific IIB subunit | 3.47 | **5.86** |
| Teth5140265 | | phosphotransferase system PTS, lactose/cellobiose-specific IIA subunit | 3.50 | **5.66** |
| Teth5140268 | | PTS system, mannitol-specific IIC subunit | 0.17 | 0.33 |
| Teth5140269 | | transcriptional antiterminator, BglG | 0.51 | 0.99 |
| Teth5140270 | | phosphoenolpyruvate-dependent sugar phosphotransferase system, EIIA 2 | -0.24 | -0.46 |
| Teth5140271 | | mannitol-1-phosphate 5-dehydrogenase | -0.96 | -1.89 |
| Teth5140533 | | phosphotransferase system, lactose/cellobiose-specific IIB subunit | -3.23 | **-5.67** |
| Teth5140534 | | PTS system lactose/cellobiose family IIC subunit | -3.95 | **-6.74** |
| Teth5140535 | | phosphotransferase system PTS, lactose/cellobiose-specific IIA subunit | -3.65 | **-6.89** |
| **B. Carbon metabolism** | | |  |  |
| Teth5140266 | | glycoside hydrolase family protein | 4.92 | **7.40** |
| Teth5140267 | | Beta-glucosidase | 4.49 | **7.14** |
| Teth5141025 | | dihydroxyacetone kinase, DhaK subunit | 1.41 | **2.54** |
| Teth5141026 | | dihydroxyacetone kinase, L subunit | 1.79 | **2.85** |
| Teth5141747 | | ribulose-phosphate 3-epimerase | -1.18 | **-2.09** |
| Teth5141923 | | transketolase domain-containing protein | 1.58 | **3.02** |
| Teth5140162 | | ribokinase | -1.34 | **-2.63** |
| Teth5140164 | | ABC transporter related | -1.53 | **-3.05** |
| Teth5140165 | | monosaccharide-transporting ATPase | -1.90 | **-3.67** |
| Teth5140166 | | monosaccharide-transporting ATPase | -1.80 | **-3.50** |
| Teth5140408 | | phosphopentomutase | -1.85 | **-2.77** |
| Teth5141600 | | phosphoglycerate mutase | -1.97 | **-2.81** |
| Teth5141416 | | 6-phosphogluconate dehydrogenase-like protein | -3.23 | **-4.45** |
| Teth5141417 | | glucose-6-phosphate 1-dehydrogenase | -1.30 | **-2.17** |
| Teth5141195 | | pyruvate kinase., pyruvate, water dikinase | -1.41 | **-2.06** |
| Teth5141081 | | 6-phosphogluconate dehydrogenase | -1.31 | **-2.04** |
| Teth5142238 | | phosphomannomutase | -1.31 | **-2.10** |
