# Supplementary material for: The Thermoanaerobacter Glycobiome Reveals Mechanisms of Pentose and Hexose Co-Utilization in Bacteria
Source: PLoS Genet. 2011 Oct 13;7(10):e1002318. doi: 10.1371/journal.pgen.1002318 (PMC3192829; doi:10.1371/journal.pgen.1002318)
Supplement: Table S10 — Up- or Downregulated Genes in Energy Metabolism (COG C) in Thermoanaerobacter sp. X514 under Cellobiose. Bold fonts indicate |Z score| ≥2. (DOC) [file pgen.1002318.s020.doc]

**Table S10. Up- or Down-regulated Genes in Energy Metabolism (COG C) for *Thermoanaerobacter* sp. X514 under Cellobiose.** Bold fonts indicated |Z score|≥ 2.

| **Gene ID** | **Annotation** | **Cellobiose vs Glucose** | |
| --- | --- | --- | --- |
|  |  | **log_2_*R*** | **Z score** |
| Teth5140079 | electron transport complex, RnfABCDGE type, C subunit | -1.91 | **-3.01** |
| Teth5140080 | electron transport complex, RnfABCDGE type, D subunit | -1.80 | **-2.61** |
| Teth5140081 | electron transport complex, RnfABCDGE type, G subunit | -2.30 | **-3.48** |
| Teth5140082 | electron transport complex, RnfABCDGE type, E subunit | -2.19 | **-3.20** |
| Teth5140083 | electron transport complex, RnfABCDGE type, A subunit | -2.47 | **-2.61** |
| Teth5140084 | electron transport complex, RnfABCDGE type, B subunit | -2.55 | **-3.75** |
| Teth5141850 | sodium ion-translocating decarboxylase, beta subunit | -2.69 | **-3.31** |
| Teth5141851 | sodium pump decarboxylase, gamma subunit | -1.13 | **-2.17** |
| Teth5142362 | V-type ATP synthase subunit D Teth5142362 | -1.41 | **-2.51** |
| Teth5140972 | Na+/H+ antiporter NhaC | -2.63 | **-4.61** |
| Teth5141571 | sodium:dicarboxylate symporter | -2.79 | **-4.91** |
| Teth5141587 | 2-oxoglutarate ferredoxin oxidoreductase subunit alpha | -1.19 | **-1.62** |
| Teth5141588 | 2-oxoglutarate ferredoxin oxidoreductase subunit beta | -1.41 | **-2.44** |
| Teth5140145 | iron-containing alcohol dehydrogenase | -2.48 | **-4.75** |
| Teth5140146 | NADH:flavin oxidoreductase/NADH oxidase | -2.67 | **-5.00** |
| Teth5140216 | L-lactate dehydrogenase | -3.55 | **-4.69** |
| Teth5140218 | dehydrogenase (flavoprotein)-like protein | -3.71 | **-7.20** |
| Teth5140219 | dehydrogenase (flavoprotein)-like protein | -3.42 | **-6.65** |
| Teth5140415 | trans-homoaconitate synthase | -2.76 | **-5.51** |
| Teth5140416 | aconitate hydratase | -2.80 | **-5.52** |
| Teth5140830 | malate dehydrogenase | -2.22 | **-4.32** |
