# Supplementary material for: The Thermoanaerobacter Glycobiome Reveals Mechanisms of Pentose and Hexose Co-Utilization in Bacteria
Source: PLoS Genet. 2011 Oct 13;7(10):e1002318. doi: 10.1371/journal.pgen.1002318 (PMC3192829; doi:10.1371/journal.pgen.1002318)
Supplement: Table S11 — Top 23 Genes with the Highest Numbers of Connections in the Thermoanaerobacter Glycobiome Network. (DOC) [file pgen.1002318.s021.doc]

**Table S11. The Top 23 Genes with the Highest Numbers of Connections in the Thermoanaerobic Glycobiome** Network.

| **Gene ID** | **No. of links** | **Annotation** | **COG** |
| --- | --- | --- | --- |
| Teth5140154 | 12 | xylulokinase | G |
| Teth5140155 | 20 | D-xylose ABC transporter, periplasmic substrate-binding protein | G |
| Teth5140157 | 28 | ABC transporter related | G |
| Teth5140268 | 24 | PTS system, mannitol-specific IIC subunit | G |
| Teth5140269 | 17 | transcriptional antiterminator, BglG | K |
| Teth5140270 | 34 | phosphoenolpyruvate-dependent sugar phosphotransferase system, EIIA 2 | G |
| Teth5140402 | 32 | ABC transporter related | R |
| Teth5140403 | 12 | inner-membrane translocator | R |
| Teth5140404 | 28 | inner-membrane translocator | R |
| Teth5140408 | 21 | phosphopentomutase | G |
| Teth5140941 | 17 | 4Fe-4S ferredoxin iron-sulfur binding domain-containing protein | C |
| Teth5140942 | 20 | 2-ketoisovalerate ferredoxin reductase | C |
| Teth5140986 | 29 | xylose isomerase domain-containing protein |  |
| Teth5140988 | 20 | oxidoreductase domain-containing protein | R |
| Teth5141589 | 26 | pyruvate/ketoisovalerate oxidoreductase, gamma subunit | C |
| Teth5141792 | 37 | oligopeptide/dipeptide ABC transporter, ATPase subunit | E |
| Teth5141793 | 34 | oligopeptide/dipeptide ABC transporter, ATPase subunit | EP |
| Teth5141794 | 29 | binding-protein-dependent transport systems inner membrane component | EP |
| Teth5141795 | 34 | binding-protein-dependent transport systems inner membrane component | EP |
| Teth5141796 | 25 | extracellular solute-binding protein | E |
| Teth5141935 | 21 | iron-containing alcohol dehydrogenase | C |
| Teth5141936 | 20 | acetate kinase | C |
| Teth5141937 | 21 | ethanolamine utilization protein-like protein | E |
| Teth5141938 | 16 | microcompartments protein | E |
| Teth5141939 | 18 | microcompartments protein | QC |
| Teth5141942 | 17 | aldehyde dehydrogenase | C |
