# Supplementary material for: The Thermoanaerobacter Glycobiome Reveals Mechanisms of Pentose and Hexose Co-Utilization in Bacteria
Source: PLoS Genet. 2011 Oct 13;7(10):e1002318. doi: 10.1371/journal.pgen.1002318 (PMC3192829; doi:10.1371/journal.pgen.1002318)
Supplement: Table S12 — Expression of the Nine Alcohol Dehydrogenase Genes from X514 under Different Carbohydrates. Bold fonts indicate |Z score| ≥2. G: glucose; X: xylose; F: fructose; C: cellobiose; Early: early exponential phase; Mid: mid exponential phase; and Late: late exponential phase. (DOC) [file pgen.1002318.s022.doc]

**Table S12. Expression of the Nine Alcohol Dehydrogenase Genes of *Thermoanaerobacter* sp. X514 under the Different Carbohydrates. Bold fonts indicated |Z score|≥2. G: glucose, X: xylose, F: fructose, C: cellobiose, Early: early-exponential phase, Mid: mid-exponential phase, Late: late-exponential phase.**

| **Gene ID** | **Annotation** | **X vs G** | | **F vs G** | | **C vs G** | | **Early vs Mid (G)** | | **Late vs Mid (G)** | | **Early vs Mid (X)** | | **Late vs Mid (X)** | |
| --- | --- | --- | --- | --- | --- | --- | --- | --- | --- | --- | --- | --- | --- | --- | --- |
|  |  | **log_2_*R*** | **Z score** | **log_2_*R*** | **Z score** | **log_2_*R*** | **Z score** | **log_2_*R*** | **Z score** | **log_2_*R*** | **Z score** | **log_2_*R*** | **Z score** | **log_2_*R*** | **Z score** |
| Teth5140241 | iron-containing alcohol dehydrogenase | 1.35 | 1.08 | -0.11 | -0.10 | -1.00 | -0.89 | -0.06 | -0.04 | 1.23 | 0.91 | **-2.77** | **-2.36** | 0.54 | 0.36 |
| Teth5140564 | iron-containing alcohol dehydrogenase | 0.28 | 0.24 | 0.10 | 0.09 | -1.58 | -1.58 | -0.31 | -0.20 | 0.77 | 0.39 | -1.75 | -1.30 | 0.86 | 0.57 |
| Teth5140627 | *adhE* | 0.06 | 0.05 | 0.47 | 0.35 | -1.08 | -0.76 | -0.71 | -0.38 | 0.84 | 0.40 | **-2.71** | **-2.52** | 0.83 | 0.51 |
| Teth5140653 | *adhB* | -0.04 | -0.04 | 0.52 | 0.83 | -0.71 | -0.95 | -0.41 | -0.26 | 0.22 | 0.12 | -1.65 | -1.41 | 0.86 | 0.52 |
| Teth5140654 | *adhA* | -0.31 | -0.28 | 0.63 | 0.50 | -0.80 | -0.72 | -1.04 | -1.87 | -0.33 | -0.58 | **-1.89** | **-2.04** | 0.56 | 0.31 |
| Teth5141882 | iron-containing alcohol dehydrogenase | 0.67 | 1.26 | -0.09 | -0.17 | **-1.34** | **-2.61** | -0.62 | -1.20 | 0.00 | -0.01 | **-2.22** | **-4.10** | 0.63 | 1.18 |
| Teth5141935 | iron-containing alcohol dehydrogenase | **1.66** | **2.64** | **1.54** | **2.56** | 0.13 | 0.21 | -0.76 | -1.16 | 0.23 | 0.36 | **-2.39** | **-3.85** | 1.42 | 1.80 |
| Teth5141808 | short-chain alcohol dehydrogenase | -0.22 | -0.39 | **-1.41** | **-2.61** | **-2.42** | **-4.39** | -0.09 | -0.15 | 0.23 | 0.37 | **-2.35** | **-4.34** | 0.31 | 0.49 |
| Teth5140145 | iron-containing alcohol dehydrogenase | -0.33 | -0.63 | -0.50 | -0.97 | **-2.48** | **-4.75** | **-1.93** | **-3.65** | **-1.99** | **-3.76** | **-1.28** | **-2.50** | 0.46 | 0.88 |
