# Supplementary material for: The Thermoanaerobacter Glycobiome Reveals Mechanisms of Pentose and Hexose Co-Utilization in Bacteria
Source: PLoS Genet. 2011 Oct 13;7(10):e1002318. doi: 10.1371/journal.pgen.1002318 (PMC3192829; doi:10.1371/journal.pgen.1002318)
Supplement: Text S1 — Supplemental Materials. (DOC) [file pgen.1002318.s025.doc]

**Text S1**

**The *Thermoanaerobacter* Glycobiome Reveals Mechanisms of Pentose and Hexose Co-Utilization in Bacteria**

**Lu Lin^1, 2, §^, Houhui Song^1, §^, Qichao Tu^2^, Yujia Qin^2^, Aifen Zhou^2^, Wenbin Liu^2^, Zhili He^2^, Jizhong Zhou^2, *^, and Jian Xu^1, *^**

^1^CAS Key Laboratory of Biofuels, Shandong Key Laboratory of Energy Genetics and BioEnergy Genome Center, Qingdao Institute of BioEnergy and BioProcess Technology, Chinese Academy of Sciences, Qingdao, Shandong, P. R. China.

^2^Institute for Environmental Genomics and Department of Botany and Microbiology, University of Oklahoma, Norman, OK, USA.

^§^These authors contributed equally to this work.

**Running title:** Co-utilization of pentose and hexose in bacteria

*****Corresponding authors

Jian Xu. Tel.: + 86 532 8066 2653; fax: +86 532 8066 2654

E-mail: [xujian@qibebt.ac.cn](mailto:xujian@qibebt.ac.cn) (Jian Xu)

Jizhong Zhou. Tel.: +01 405 325 6073; fax: +01 405 325 7552

Email: [jzhou@ou.edu](mailto:jzhou@ou.edu) (Jizhong Zhou)

**Part I. *Thermoanaerobacter* sp. X514 Efficiently and Simultaneously Metabolizes both Hexoses and Pentoses but with Distinct Product Portfolios.** Glucose, xylose, and cellobiose are the primary mono- and disaccharides released from lignocellulose, while fructose, the major component of fructans, enabled the highest growth rate in X514. Therefore, growth experiments were first performed in defined medium with glucose, xylose, fructose and cellobiose as the sole carbon source. The results of these experiments suggest that different carbon substrates result in distinct product portfolios. First, more fructose than any other carbohydrate was consumed, and fructose resulted in more ethanol, acetate and lactate production. Therefore, under fructose, more carbon fluxes were directed to fermentation pathways (**Figure S1A** and **Figure S1B**). Second, between glucose and xylose, no significant difference (*p* > 0.05) was observed in ethanol yield and substrate consumed (**Figure S1A** and **Figure S1B**), though xylose yielded less lactate. Altogether, given the lower number of carbons in xylose than in glucose, the conversion efficiency from xylose to ethanol was higher than for glucose. Third, when compared with glucose, cellobiose produced less acetate. Because acetate production is a key step in ATP generation, cellular ATP production was less efficient under cellobiose than under glucose, which was consistent with the slower growth under cellobiose (**Figure 1A** and **Figure S1E**).

In addition, to appreciate the dynamics of carbon utilization in X514, the complete time-course of carbon consumption was investigated. A relative delay (“xylose lag”) was observed when compared to glucose (**Figure S1D**).

**Part II. Diversity of Nodes and Modules in the *Thermoanaerobacter* Glycobiome Network.** The functional network serves as an experimental foundation for annotating and validating the structure and function of genes, operons, novel regulatory circuits and other genomic features because similar gene-expression patterns suggest functional relationships [1].

Among the 614 total nodes (genes) included in the network, there are 120 hypothetical proteins, suggesting a much broader scope of the glycobiome than those deduced solely on the basis of genome annotation. For example, the hypothetical proteins (encoded by *teth5141834-1835*) in Module 8 (mostly related to xylose catabolism) strongly correlate with carbohydrate transport (*teth5140269-0272* and *teth0154-0157*), dipeptide ABC transport (*teth5141792-1795*) and energy production (*teth5141589*, *1935*, *1914*, and *0945*) genes via multiple links, thereby serving as junctions between carbohydrate (likely xylose-specific) transport and energy production (**Figure S2A**). Another example is the hypothetical proteins found in Module 4 (mostly involved in cellobiose catabolism or encoding hypothetical proteins). They include Teth5140704-0709, which are directly linked to the XRE family transcriptional regulator (Teth5140703) and other hypothetical proteins (Teth5140710-0713, 0725, 0726-0729, 0733-0734 and 0736-0737) (**Figure S2B** and **Table S2**). Notably, all of these genes are upregulated in the presence of cellobiose, suggesting previously unknown members of the cellobiose-specific glycobiome.

A total of 115 partial (consisting of at least two genes) or complete operons, which were previously only predicted and not experimentally tested [2], are found in the network (**Figure 3A**). For 90 of the predicted operons, member genes all reside in a single module, thus validating the previous definition of these putative operons. For the additional 24 predicted operons, member genes are divided into separate modules. However, they are all connected by the links between modules, underscoring the different functional role that each member of the putative operon contributes and the role of the predicted operon in integrating multiple functions into a coordinated cellular activity. However, one inconsistency with the original putative operon structures is discovered. The components of this predicted operon (*teth5140263-0271*; defined according to Microbes Online [2]) are not found in a single module: one part, which encodes a mannitol-specific PTS, resides in Module 8 (*teth5140268-0271*), whiles the other part, encoding a cellobiose-specific PTS (*teth5140263-0267*), resides in Module 4 (**Figure 3B** and **Figure S2B**). Additionally, there are no links between them (**Figure 3A**). Thus, this example highlights the value of the network in uncovering predicted operon structure annotation errors or alternative regulatory mechanisms.

The co-expression network also reveals novel regulatory units in *Thermoanaerobacter* via its component nodes and modulized structure [3]. One such example involves *teth5141567*, which is annotated as a transcriptional regulator, but neither its functional role nor its regulatory targets were clear. The network reveals that this regulator positively correlates with an RNA-binding S1 domain-containing protein (Teth5141607) and a translation initiation factor IF-3 (Teth5142054), thus suggesting its role and target genes in regulating translation (**Figure S3D**). In another case, the regulatory protein ArsR (Teth5141161 and 1275), which functions as a transcriptional repressor of an arsenic resistance operon in other bacteria [4], might be responsible for the regulation of heavy metal translocating P-type ATPase (*teth5141162-1163* and *teth5141276*) in X514 because it directly connects to those genes (**Figure S3D**).

**Part III. Additional Genes Found in the Module 8 (Mostly Xylose Utilization and Energy Production Genes).** First, genes encoding ABC transporters are found in Module 8 (**Figure 3B**). These include xylose-specific ABC transporters in COG G (*teth5140155-0157* and *teth5140989*), oligopeptide ABC transporters in COG E (*teth5141792-1793*), an ABC inorganic iron transport system in COG P (*teth5141395-1398* and *1793*), an ABC transport system in COG O and COG R (*teth5140402* and *1396*), and an additional ABC transport system (*teth5140402-0404*). This finding reveals that genes encoding ABC transporters tend to be under “central” control because their expression is tightly coordinated, independent of their substrates. In contrast, genes encoding carbohydrate PTSs, which are found in a variety of modules (**Figure 3A**), feature “de-centralized” regulation.

Second, a B_12_-dependent propanediol utilization pathway (COG Q) is present. All these genes (*teth5141947-1948*) are upregulated during xylose fermentation. Because propanediol could be used as an alternative source for ATP, this finding provides further evidence that ethanol and energy generation is more active in the presence of xylose.

Third, two previously unrecognized regulatory sub-modules that are involved in xylose utilization are detected. Besides the sub-module of BglG (see **Main Text**), another such sub-module centers on the RpiR-family transcriptional regulator Teth5141590 (**Figure S3C**). Members of this family are regulators of phosphosugar metabolism genes [5]. Thus, *teth5141590* is co-expressed with the other members of the phosphosugar metabolism locus (*teth5141589-1591*). However, the network reveals a positive correlation between this regulator and genes involved in microcompartment systems (*teth5141951-1955* and *teth5141938-1939*), which protect the cell from damage by organic metabolites [6]. Thus, these findings suggest a novel functional association between phosphosugar metabolism and cell protection machineries and identify the key regulatory role of RpiR in this crosstalk.

**Part IV. Module 7 (Mostly Fructose Utilization Genes)**. A module of fructose metabolism is identified in the network (**Figure S5A**). These genes are generally induced by fructose. They include a fructose-specific PTS (*teth5140823-0826* and *0577-0578*) and proteins required for fructose metabolism (*teth5140575-0576*) (**Table S6**). In addition, the locus (*teth5141853-1855*) involved in vitamin B_12_-dependent methylmalonyl-CoA mutase (MCM) is also found in this module, which is upregulated in the presence of fructose (**Table S7**). MCM catalyzes the isomerization of methylmalonyl-CoA to succinyl-CoA in the catabolism of propanoate, which can be used as the carbon source to produce acetate and ethanol [7].

Further analysis reveals that many genes involved in energy production and conversion are upregulated under fructose (using glucose as a baseline). Examples include acetate kinase (*teth5141936*), iron-containing alcohol dehydrogenase (*teth5141935*), propanediol utilization protein (*teth5141947-1948*) and the vitamin B_12_-dependent ethanolamine utilization pathway (*teth5141937-1939* and *1945-1946*) (**Table S7** and **Table S8**). In addition, the expression of ferredoxin-NADP^+^ reductase genes (*teth5140502* and *0560*) is elevated under fructose, thereby providing reducing equivalents to ethanol fermentation (**Table S8**) [8]. Together, these results reveal the mechanism contributing to the higher levels of acetate and ethanol produced under fructose (**Figure S1B**).

**Part V. Module 4 (Mostly Cellobiose Utilization Genes and Hypothetical Genes)**. Genes in this module are induced by cellobiose (**Table S9** and **Figure S2B**). These genes include the PTS lactose/cellobiose family EIIA/B/C, which transports cellobiose across the membrane, and glycoside hydrolase (*teth5140262-0267*), which hydrolyzes cellobiose into glucose-1P and fluxes into glycolysis and the pentose phosphate pathway (PPP).

Many genes involved in energy production/conversion are downregulated under cellobiose (using glucose as a baseline). A prominent cluster is the electron-transporting, ferredoxin-linked, Na^+^-translocating Rnf complex (*teth5140079-0084*) [9]. Other examples include the Na^+^-translocating decarboxylase (*teth5141850-1851*) and the Na^+^/H^+^ antiporters (*teth5140972*), which maintain H^+^ and Na^+^ gradients, as well as Na^+^/solute symporters (*teth5141571*), which allow for the influx of solutes (**Table S10**). This finding indicates that the energy conversion from cellobiose in X514 is less efficient, consistent with the observed slower growth, lower biomass and fewer end products observed with cellobiose as the sole carbon-source (**Figure 1** and **Figure S1**). Because cellobiose is composed of two glucoses, the activity of cellobiose-specific glycoside hydrolases is probably a crucial bottleneck in the efficient utilization of disaccharides under CBP conditions.

**Part VI. Additional Modules Involving Carbohydrate Metabolism**. First, a novel energy driver of the glycobiome is identified by the presence of Module 9 which includes V-type ATPase (which is mostly found in archaea and eukaryotes) in the network instead of the F-type ATPase that functions in most bacteria [10]. Second, the presence of Module 6, which includes genes responsible for heavy metal translocation, emphasizes the importance of heavy metal ions, such as cobalt for B_12_ biosynthesis and iron for alcohol dehydrogenase, in supporting the *Thermoanaerobacter* glycobiome. Third, valine, leucine and isoleucine make a glucose-specific contribution to the glycobiome because Module 12, consisting of genes synthesizing these three amino acids (*teth5140012-0018*), is induced by glucose but not by other substrates. Therefore, a larger carbon flux is directed to amino acid biosynthesis from pyruvate during glucose fermentation (**Table S5**), explaining the observation that acetate and ethanol yields under glucose are lower than that under fructose, which is also a hexose.

**Part VII. Glucose-Specific and Xylose-Specific Genes Are Simultaneously and Actively Expressed under the Co-Presence of Glucose and Xylose**. Among the notable genes involved in COG G, COG C and COG E, the expression pattern under dual carbohydrate sources displays distinct and linked characteristics in contrast to sole carbon sources (mid exponential under the dual carbohydrate was defined as treatment with those under single glucose or xylose as controls). First, the expression of glucose- and xylose-specific transport systems was induced under dual carbohydrate treatment. In contrast to glucose as the sole carbon source, most genes in the putative operons involved in xylose uptake and transport (*teth5140153-0155* and *teth5140157-0159*) were significantly upregulated, whereas the genes involved in the glucose PTS (*teth5140412-0414*) were downregulated (**Table S3** and **Figure S6**). However, when compared with xylose as the sole carbon source, the genes encoding the glucose-specific PTS EII and its regulatory factor (*teth5140412* and *0414*) were upregulated, while xylose ABC transporter genes were downregulated. Therefore, under the co-presence of glucose and xylose, the two sets of glucose-specific and xylose-specific genes are simultaneously and actively expressed, though the fold-change of transcript abundance is less than that under each single substrate, possibly due to the need to maintain energy balance.

Secondly, under the dual carbohydrate program, the expression pattern of COG C (energy-conversion) genes was similar to the profile induced by glucose alone but not to xylose alone (**Table S4).** Therefore, the dual carbohydrate transcriptional program inherits certain features in energy conversion from the glucose-alone program.

Third, the dual carbohydrate transcriptional program maintained the distinct and crucial characteristic of activated arginine metabolism. Similar to under xylose, arginine metabolism genes (**Table S5**) remained upregulated under the dual sugars (compared to glucose alone), though the induced level was lower than that under xylose alone. Furthermore, under glucose plus xylose, few genes with altered expression were detected when compared to xylose (|Z score| < 2). In addition, genes involved in valine and leucine biosynthesis were repressed under dual carbohydrate in contrast to glucose-alone growth (**Table S5**). Hence, the dual carbohydrate transcriptional program includes features from the xylose-alone program, particularly those related to amino acid metabolism (**Table S5**).

**Part VIII. The Differentiated Roles of *adh*s in *Thermoanaerobacter***. The gene co-expression network reveals the various roles of the *adh*s. First, all the three NADPH-dependent *adh*s (*adhA/B/E*) are absent from the network. Furthermore, the expression of those genes remains constant among the carbohydrates tested. Considering that AdhB and AdhE primarily function in ethanol production from acetyl-CoA and acetaldehyde and that AdhA mediates ethanol consumption for NAD cofactor recycling [11, 12], these three Adhs in X514 may maintain basic ethanol production under different carbon substrates. Second, although the additional iron-containing *adh* (*teth5140145*) is absent from the network, it is downregulated under cellobiose, suggesting that iron-containing *adh*s are more sensitive to changes of carbohydrate substrates. Third, three iron-containing *adhs* exist in the network, including *teth5140241* and *teth5141882* in Module 1 and *teth5141935* in Module 8 (**Figure S5B and Figure S5C**). Module 1 and the genes directly linked to *teth5140241* and *teth5141882* are involved in DNA replication or modification (e.g., methylation) (**Figure 3A**), excluding the roles of these two *adhs* in energy production.

**References**

1. Stuart JM, Segal E, Koller D, Kim SK (2003) A gene-coexpression network for global discovery of conserved genetic modules. Science 302: 249-255.

2. Alm EJ, Huang KH, Price MN, Koche RP, Keller K, et al. (2005) The MicrobesOnline Web site for comparative genomics. Genome Res 15: 1015-1022.

3. Yang Y, Harris DP, Luo F, Xiong W, Joachimiak M, et al. (2009) Snapshot of iron response in *Shewanella oneidensis* by gene network reconstruction. BMC Genomics 10: 131.

4. Wu J, Rosen BP (1991) The ArsR protein is a trans-acting regulatory protein. Mol Microbiol 5: 1331-1336.

5. Kim C, Song S, Park C (1997) The D-allose operon of *Escherichia coli* K-12. J Bacteriol 179: 7631-7637.

6. Stojiljkovic I, Baumler AJ, Heffron F (1995) Ethanolamine utilization in *Salmonella typhimurium*: nucleotide sequence, protein expression, and mutational analysis of the cchA cchB eutE eutJ eutG eutH gene cluster. J Bacteriol 177: 1357-1366.

7. Bobik TA, Havemann GD, Busch RJ, Williams DS, Aldrich HC (1999) The propanediol utilization (pdu) operon of *Salmonella enterica* serovar typhimurium LT2 includes genes necessary for formation of polyhedral organelles involved in coenzyme B-12-dependent 1,2-propanediol degradation. J Bacteriol 181: 5967-5975.

8. Feng X, Mouttaki H, Lin L, Huang R, Wu B, et al. (2009) Characterization of the central metabolic pathways in *Thermoanaerobacter* sp. strain X514 via isotopomer-assisted metabolite analysis. Appl Environ Microbiol 75: 5001-5008.

9. Speelmans G, Poolman B, Abee T, Konings WN (1994) The F- or V-type Na(+)-ATPase of the thermophilic bacterium *Clostridium fervidus*. J Bacteriol 176: 5160-5162.

10. Seedorf H, Fricke WF, Veith B, Bruggemann H, Liesegang H, et al. (2008) The genome of *Clostridium kluyveri*, a strict anaerobe with unique metabolic features. Proc Natl Acad Sci U S A 105: 2128-2133.

11. Burdette D, Zeikus JG (1994) Purification of acetaldehyde dehydrogenase and alcohol dehydrogenases from *Thermoanaerobacter ethanolicus* 39E and characterization of the secondary-alcohol dehydrogenase (2 degrees Adh) as a bifunctional alcohol dehydrogenase—acetyl-CoA reductive thioesterase. Biochem J 302 ( Pt 1): 163-170.

12. Pei J, Zhou Q, Jiang Y, Le Y, Li H, et al. (2010) *Thermoanaerobacter* spp. control ethanol pathway via transcriptional regulation and versatility of key enzymes. Metab Eng 12: 420-428.
